# Narrative and Non-Narrative Discourse Skills in ADHD Across the Lifespan: A Systematic Review of the Literature

**DOI:** 10.1177/10870547251389329

**Published:** 2025-11-18

**Authors:** Elizabeth Hill, Robert Wells, Wai Chen

**Affiliations:** 1 Curtin University, Perth, WA, Australia; 2 University of Western Australia, Perth, Australia; 3 Murdoch University, Perth, WA, Australia; 4 Naresuan University, Tha Pho, Phitsanulok, Thailand

**Keywords:** ADHD, discourse, language, narrative, non-narrative

## Abstract

**Objective:**

Discourse-level language abilities are critical for successful participation in social, academic, and vocational pursuits. These abilities encompass both narrative and non-narrative genres, each serving distinct communicative functions. Narrative discourse involves spoken accounts of events or experiences, typically with a setting, characters, and a sequence of actions. Non-narrative discourse includes genres like explanations, arguments, and descriptions that convey information or ideas without a temporal structure. The aim of this review was to synthesise extant literature on discourse abilities of children and adults with ADHD across these genres.

**Methods:**

Systematic searches were conducted via CINAHL, PsycINFO, Medline, and ProQuest. The review adhered to PRISMA guidelines and was registered with PROSPERO [CRD 42022377007].

**Results:**

Thirty-nine studies were included in our review. Most studies investigated the narrative abilities of children with ADHD. ADHD was associated with atypical verbal output, characterised by atypical brevity and verbosity, dysfluency, reduced syntactic complexity, and grammatical errors. Individuals with ADHD produced fewer pronouns and conjunctions. Additionally, their discourse was less coherent and included more frequent topic changes. Similarly, speakers with ADHD omitted critical components of discourse genres. The effect of ADHD on discourse varied between adults and children with ADHD and was evident in both narrative and non-narrative discourse.

**Conclusion:**

Published evidence to date indicates that ADHD affects micro-linguistic to super-structural discourse features in children and adults, likely impacting communication success in social and academic environments. Assessing the structure and content of narrative and non-narrative genres should form routine functional evaluation in ADHD for adults and children. More research is indicated given current major gaps in areas reviewed.

## Introduction

ADHD is a common neurodevelopmental disorder characterised by age-inappropriate levels of inattention, hyperactivity, and impulsivity that impair daily functioning (American Psychiatric Association [APA], 2022). The estimated worldwide pooled prevalence of ADHD is 5.29% for those 18 years or younger (Polanczyk et al., 2007); in adults, the pooled prevalence varies from 2.58% for the persistent form of adult ADHD to 7.67% for the symptomatic form (Song et al., 2021). In clinical settings, the prevalence of ADHD is much higher, with a pooled prevalence of 32% in children and 21% in adult clinical populations (Johnson et al., 2025). The literature on language difficulties in children with ADHD is limited, yet this population is estimated to be at a threefold increased risk of co-occurring language difficulties, including *clinical* language disorder (i.e., “developmental language disorder”; Johnson et al., 2025; Korrel et al., 2017; Méndez-Freije et al., 2023; Redmond, 2004). Some evidence suggests that language difficulties among children with ADHD can persist even after controlling for co-occurring language disorders (Oram et al., 1999), indicating that communication difficulties may, in some cases, be more closely linked to the cognitive variability characteristic of ADHD rather than stemming solely from underlying language impairments (Méndez-Freije et al., 2023). However, these pathways are not necessarily mutually exclusive. The language difficulties observed in ADHD (e.g., disrupted pragmatics), could reflect an interaction between structural language impairments and variable cognitive profiles, compounding the individual’s overall language and communication abilities. Compared to children, communication difficulties in adult ADHD are underexplored in the literature. Adults with ADHD have reported less social competency than their peers (Barkley et al., 2006), and research suggests that difficulties with social skills and interpersonal relationships may be attributable to pragmatic language problems (Nilsen et al., 2013). Indeed, pragmatic language has increasingly become a central focus of research in mental health disorders (Li et al., 2017; Pawełczyk et al., 2020). For ADHD, two core symptoms of the condition (i.e., talking excessively or difficulty awaiting conversational turns) are synonymous with pragmatic language problems in the absence of clinical language disorders (Green et al., 2014).

Recently, Carruthers et al. (2021) synthesised the literature on pragmatic language impairments in children with ADHD, confined to studies of narrative storytelling and social skills; and reported more inappropriate initiation of communication, as well as poorer conversation and narrative skills in those affected (Carruthers et al., 2021). Narrative tasks^1^ have long been a preferred method of assessing verbal pragmatic language and functional communication in populations at risk of language difficulties (Botting, 2002; Hill, Claessen, et al., 2021; Westerveld & Claessen, 2014). Narrative is a form of discourse-level language; and “discourse” refers to the use of connected sentences to communicate a particular intent or purpose essential for social, academic, and vocational participation (Staikova et al., 2013). Discourse assessment evaluates an individual’s integration of language and cognitive skills required for daily communication and is a critical supplement to omnibus standardised language assessments (e.g., the Clinical Evaluation of Language Fundamentals – Fifth edition [CELF-5]; Wiig et al., 2013), which assess discrete language functions (e.g., grammar and phonological awareness). Indeed, discourse assessment (particularly narrative assessment) is included in contemporary best practice guidelines for various clinical populations to counteract the limited ecological validity of omnibus standardised tests (Ebert & Scott, 2014).

While previous reviews have offered important insights into the pragmatic and narrative discourse challenges faced by children with ADHD, their scope was limited in two key respects. First, both focussed predominantly on narrative discourse, despite effective communication across childhood, adolescence, and adulthood, relying on a broader range of discourse types, including procedural, expository, persuasive, and conversational forms (Hill, Claessen, et al., 2021; Hill, Whitworth, et al., 2021). Examining language use across these genres is essential, as each serves distinct social, academic, and vocational functions and imposes different cognitive and linguistic demands (Hill et al., 2018). For example, narrative discourse may appear relatively strong in individuals with ADHD due to early and frequent exposure in childhood and school settings, which supports familiarity with its structure and reduces cognitive load (Hill, Whitworth, et al., 2021). Consequently, more cognitive resources can be directed towards maintaining topic and coherent output. In contrast, persuasive and expository tasks are considered more complex, drawing on higher-order cognitive and linguistic skills, including perspective-taking and executive functioning, to construct arguments and convey abstract ideas (Nippold, 2007). As such, individuals with ADHD may perform differently across different discourse genres.

Second, neither study provides insight into discourse in adolescents or adults with ADHD. While both studies included participants up to 18 years, they reviewed limited literature on discourse-level language in the adolescent years (Carruthers et al., 2021; Jepsen et al., 2022). Indeed, evidence indicates that the communicative demands and contexts of adolescent and adult life are substantially different from those of childhood. For instance, while young children’s discourse is often assessed in academic contexts, adolescent and adult discourse is more likely to be functional, transactional, or related to vocational and interpersonal responsibilities (e.g., explaining processes at work, managing conflict, and developing complex relationships; Dipper & Pritchard, 2017). This shift in communicative context may mean that the manifestation of discourse difficulties also changes across development. Moreover, developmental changes in cognition may interact with ADHD symptoms in older adolescence and adulthood in ways that differentially affect discourse (Hill et al., 2018; Marini et al., 2025). For example, age-related changes in cognition during adulthood, such as gradual declines in working memory and executive functioning, may interact with ADHD symptoms to affect discourse production. These interactions could lead to different patterns of discourse difficulties compared to those observed in younger childhood, with potential implications for coherence, organisation, and self-monitoring in spoken language (Hill et al., 2018; Marini et al., 2025). Consequently, it cannot be assumed that the discourse difficulties observed in younger children with ADHD will persist unchanged throughout adolescence and adulthood, nor that the same assessment approaches will adequately capture them (Kemper, 2006). A review that takes a lifespan approach is therefore needed to examine these potential differences and address the current gap in lifespan perspectives on discourse in ADHD.

Understanding the impact of ADHD on discourse production skills is highly relevant to clinical practice in mental health. Discourse skills are involved in the provision of a psychiatric history or an account of facts, describing a process involved in therapy or medication, negotiating treatment options, and engaging with psychological therapy (Hobson et al., 2022). Carruthers et al. (2021) conducted the first systematic review on pragmatic language, including studies of conversational and narrative discourse skills in children with ADHD. Jepsen et al. (2022) extended the work of Carruthers et al. (2021) and focussed specifically on the nature of narrative discourse in children with ADHD, adding a detailed account of the nature of storytelling skills in this population. Following their review of 16 studies, Jepsen et al. (2022) corroborated the findings of Carruthers et al. (2021) and concluded that children with ADHD produced shorter, less coherent, and more poorly organised discourse relative to neurotypical peers.

As both Carruthers et al. (2021) and Jepsen et al. (2022) only examined studies of children with ADHD, and had a dominant focus on narrative discourse, we argue that this provides a limited understanding of the influence of ADHD on discourse. Indeed, there are multiple genres of discourse that children *and* adults are required to use in social, academic, vocational, and clinical settings. There are five common genres of discourse used in daily communication (see Table 1 for definitions), including (i) narrative; (ii) procedural; (iii) expository; (iv) persuasion; and (v) conversation (Hill, Claessen, et al., 2021). We consider it important to synthesise extant literature across all genres to develop a detailed understanding of the influence of ADHD on discourse skills in children and adults. There are however certain challenges to doing this.

**Table 1.** Descriptions and Examples of Everyday Discourse Genres.

Genre	Purpose	Description	Use in mental health context
Narrative	To tell a story (factual or fictional)	Narrative follows a stereotypical structure, starting with characters, time and location, followed by actions and events, leading to a climax and then resolution, all of which combine to convey the core message of the story.	Recounting personal experiences (e.g., traumatic events or previous intervention)
Procedure	To communicate a process	Procedures consist of aim, materials, and steps as a protocol directed towards achieving a specific goal	Describing an understanding of procedural steps involved in medication or treatment compliance
Expository	To describe, inform, analyse	Expositions contain evidence, beliefs, and descriptions of ideas, concepts, people.	Descriptions of feelings and analyses of causal relationships between action and consequence (e.g., emotions triggered by particular thoughts or behaviours)
Persuasion	To convince the listener to adopt a particular opinion/position	Persuasive discourse typically includes statements of opinion, followed by key arguments and supporting evidence. It requires the speaker to adopt, and ultimately shift, the listener’s opinion drawing on perspective taking and complex language.	Argument and persuasion (e.g., explanation of why you feel the way you feel, or why others would or should feel a particular way);
Conversation	To share ideas and build relationships, between two or more speakers	Conversation can involve rapid shifts between multiple genres. Conversation is linguistically and cognitively complex, as speakers and listeners have to track and manage all verbal and non-verbal cues in real time, with rapid unfolding of contents, ideas, and emotions.	Clinical dialogue. Development of therapeutic alliance/rapport building between consumer and clinician.

*Source*. Hill, Claessen, et al. (2021), Stein and Glenn (1979), and Turkstra (2000).

Historically, clinicians and researchers have utilised different methods to assess and characterise discourse abilities across these five different genres. As such, the characterisation of discourse in ADHD is complex (Jepsen et al., 2022) as discourse production is underpinned by many functions that are commonly affected by this disorder, such as pragmatics, attention, memory, and executive function (Tannock & Schachar, 1996). Jepsen et al. (2022) highlighted inconsistencies in the terminological and methodological characterisation of discourse within the ADHD narrative literature; and such inconsistencies pose a barrier to synthesising existing studies. This barrier has been reported in other neurotypical (Hill, Claessen, et al., 2021) and clinical populations (e.g., acquired brain injury; Bryant et al., 2017), but it has been overcome by adopting more recent systematic frameworks to identify, label, and describe multiple features of the content and structure discourse output. This innovative framework (explicated below) provides us an opportunity to conduct a more comprehensive systematic review here to address the gaps in the literature.

More specifically, the analysis framework typically utilises four-levels of measurement^2^ (Power et al., 2020; while exact terms may vary) : (i) micro-linguistic (i.e. within clauses), (ii) macro-linguistic (i.e., between clauses), (iii) macro-structural (i.e., capturing the relevance of topics and themes across multiple clauses), and (iv) super-structural (i.e., whole text; a detailed description of these levels is provided in Supplemental Material). Each level examines a different aspect of discourse content or structure. It is recommended that discourse abilities are characterised at each level to provide the most complete picture of language capacity.

To our knowledge, this novel framework has not been applied to discourse analysis and integration in ADHD, representing a current gap in the literature. Moreover, the extant literature appears to be skewed towards profiling narrative discourse in children with ADHD to our knowledge, and existing literature on discourse in adults with ADHD has not been considered in past reviews. Furthermore, there has been limited application of standardised terms (such as the four-levels of measurement mentioned above) to identify and describe the impact of ADHD on discourse across the lifespan (Hill, Claessen, et al., 2021). While previous reviews have synthesised the literature on pragmatic skills and narrative discourse only in *children* with ADHD (Carruthers et al., 2021; Jepsen et al., 2022), there has yet to be a systematic synthesis of discourse in ADHD that (a) considers both children *and* adults with the disorder and (b) considers non-narrative genres. Consequently, we address these gaps in the literature by applying the four-level measurement framework (i.e., micro-linguistic to super-structural features) to systematically describe studies of narrative *and* non-narrative discourse abilities in both children *and* adults with ADHD.

## Method

This review was conducted in accordance with the Preferred Reporting Items for Systematic Reviews and Meta-Analyses (PRISMA) Checklist (Moher et al., 2009) to identify relevant studies of discourse-level language abilities of speakers with ADHD. This review is registered with the international Prospective Register of Systematic Reviews (PROSPERO; registration number: CRD 4202237707).

### Eligibility Criteria

Studies were eligible for inclusion if participants were reported to have a confirmed diagnosis of ADHD. Studies were not eligible for inclusion if participants were in the process of being assessed for ADHD. No restrictions were placed on participant age or ADHD-related factors such as “subtype” (or “presentation”) or “severity.” As in similar reviews (e.g., Jepsen et al., 2022), studies were eligible if they reported direct elicitation and linguistic analysis of spoken discourse output in participants with ADHD. Studies that assessed only discrete language functions (e.g., grammar and phonological awareness) using standardised (e.g., CELF-5; Wiig et al., 2013) or non-standardised tasks were not eligible for inclusion unless they also elicited and analysed samples of spoken discourse. Studies were excluded if participants had additional co-occurring diagnoses that may affect language skills (e.g., autism spectrum disorder or language disorder). Studies that included participants with ADHD and a co-occurring diagnosis were eligible for inclusion only if they also included a group of participants with ADHD-only. Inclusion of a control or comparison group was not an eligibility requirement; therefore, no inclusion or exclusion criteria were applied to control or comparison participants. The search was limited to published, peer-reviewed, full-text sources reporting empirical data, written in English, with no restrictions placed on the study design or location.

### Search

Four electronic databases, CINAHL, PsycINFO, Medline, and ProQuest were searched initially in November 2023. A follow-up search was conducted in November 2024 to capture sources that had been published since initial searching. Key and MeSH heading terms were truncated, exploded, and adjusted, and included ADHD and discourse-related terms informed by relevant previous reviews and original studies (Carruthers et al., 2021; Hill et al., 2018; Hill, Claessen, et al., 2021; Jepsen et al., 2022). To locate studies related to ADHD, terms included: attention deficit hyperactiv*, “ADHD,” “ADD,” “ADDH,” attention deficit*, attention disorder*, inattentiv*, hyperactive*, “HKD,” and hyperkine*. To identify studies of discourse abilities across a range of everyday genre, search terms included discourse, verbal behavio?r, conversat*, monologue*, persuas*, procedur*, exposit*, argument*, narrat*, retell*, recount*, and story*. These terms were selected based on a definition of discourse as the production of spoken output (longer than a single sentence) used for a specific social, academic, or vocational function (Dipper & Pritchard, 2017). Specific terms were also selected based on previous reviews of discourse in ADHD (Carruthers et al., 2021; Jepsen et al., 2022).

#### Screening and Study Selection

**Figure 1.**
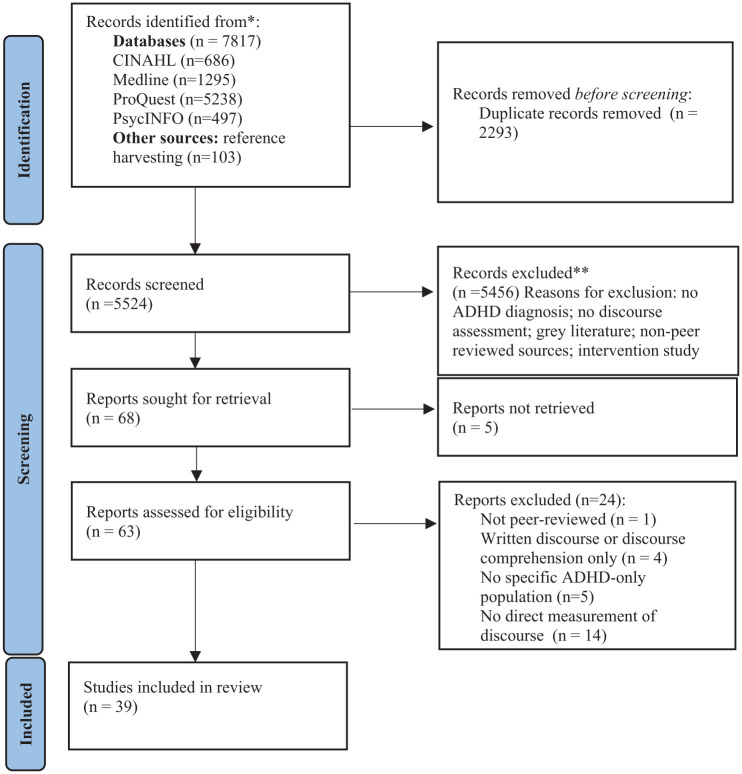
PRISMA flowchart on the different phases of the systematic review (from: Page et al., 2021).

Initial database searching yielded a total of 7,817 publications (*n* = 2,293 duplicates). Reference harvesting of relevant peer-reviewed articles identified a further 103 articles. Before title and abstract screening, authors piloted eligibility criteria. Guided by best-practice recommendations, two authors independently applied inclusion criteria to a random selection of 30 titles and abstracts to determine criteria clarity and appropriateness (Polanin et al., 2019). This resulted in 100% agreement between the authors. The remaining titles and abstracts (*n* = 5,524) were screened for eligibility for inclusion by two reviewers (*EH, RW*), with 96% agreement on papers meeting inclusion criteria. Where present, discrepancies in reviewer decisions were due to terminology related to ADHD and spoken discourse assessment. Differences were resolved following discussion, and 36 articles were subsequently excluded, resulting in 68 studies meeting the criteria for full-text review. A total of 5,252 studies were excluded due to not meeting inclusion criteria (see reasons for exclusion in Figure 1). During the subsequent stage, five studies were excluded due to unavailability (i.e., full-text not available in English, or access to full-text unavailable). Twenty-four papers were excluded because of failure to meet inclusion criteria following full-text review. A final total of 39 studies were included in the current review (these studies are marked with an asterisk [*] in the reference list).

#### Quality Assessment

This review aimed to descriptively map reported discourse features and patterns across studies rather than to estimate intervention effects. Given the narrative synthesis approach and heterogeneity in methodology and reporting across studies, formal appraisal of the certainty of evidence (e.g., consistency, precision, and directness) was not undertaken. The quality of the included studies was independently assessed by two authors (redacted) using the Standard Quality Assessment Criteria for Evaluating Primary Research Papers (Quantitative Studies; Kmet et al., 2004). This tool consists of a 14-item checklist evaluating key aspects of each study, including the clarity of the research question or objective, recruitment and group selection methods, outcome measures, and analytic procedures, among other elements (Kmet et al., 2004). Study quality was scored as very strong (90%–100%), strong (70%–89%), adequate (50%–69%), or limited (<50%). Studies are assessed on In cases of disagreement, details were discussed until a consensus was reached.

#### Charting the Data and Data Synthesis

Two authors (*redacted*) undertook full-text review and data extraction using an electronic extraction form. Data from eligible studies were extracted according to the following categories: study characteristics (author, year, and study site), study design, aim(s), sample characteristics, discourse assessment and analysis methods, and main findings. Given the anticipated methodological heterogeneity across included studies, particularly in discourse elicitation tasks, outcome measures, sample characteristics, and reporting practices, a meta-analysis was not conducted. A narrative synthesis was conducted to integrate findings across diverse study designs and outcomes. This approach was well suited to the review’s aims of mapping discourse characteristics in individuals with ADHD and identifying gaps in the evidence base (Higgins et al., 2022). Synthesis and reporting were conducted following the SWiM (Synthesis Without Meta-analysis) reporting guidelines (Campbell et al., 2020). See Supplemental Material for completed SWiM checklist.

## Results

### Study Characteristics

This review included 39 quantitative studies published between 1983 and 2024. Most publications originated from the United States (*n* = 19), followed by Canada (*n* = 3), the Netherlands (*n* = 3), Brazil (*n* = 3), Sweden (*n* = 2), and the UK (*n* = 2). The remaining seven were comprised of one study each from Australia, Denmark, Germany, Greece, South Korea, South Africa, and Spain.

### Quality Assessment

Overall quality rating for the included articles was high, with 24 of the 39 articles being rated as very strong, reflecting a quality rating percentage of 90% or higher (see Table 2). Common strengths across papers included detailed information regarding methodology, analysis, and results, while weaker studies did not adequately detail their subject selection. A detailed breakdown of the quality assessment data can be found in Supplemental Table 1.

**Table 2.** Study and Participant Characteristics.

Author (year), study site	Design	ADHD sample								Control sample		Qual (%)
		Age *M* (*SD*)	*N* (%male)	Med.	Pres. (*n*)	IQ *M* (*SD*)	Oral language ability^a^			*N*	Age *M* (*SD*)	
							Test (s)	Domain	Descriptor			
Child studies												
Baixauli-Fortea et al (2018), Spain	Cohort	9.14 (1.41)	35 (91)	Yes	x	K-BIT 99.03 (9.87), Comparable to controls	WISC-IV	Vocabulary	Below controls	37	8.54 (1.26)	95
Bangert and Finestack (2020), USA	Within-subjects	9.3 (2.6)	25 (68)	x	x	DAS-II NVIQ, 96.81 (14.07) VIQ 91.92, (13.87)	CELF-5 RS	Expressive Lang	Within normal limits	No	n/a	82
Barkley et al (1983), Canada	Cohort	9.2 (1.8)	18 (100)	Mixed	x	x	PPVT	Verbal IQ	Comparable to controls	18	8.59 (1.24)	89
Bergman and Hallin (2021), Sweden	Cohort	13.6 (1.3)	15 (53)	Mixed	x	≥70 (test n/r)	TROG 2, CELF-4 RS	Grammar; Expressive language	Below controls	31	13.7 (1.2)	100
Boo et al (2022), USA	Cohort	11.9 (2.2)	24 (88)	x	x	WASI-II, FSIQ 96.29 (16.1)	WASI-II	VIQ	Below controls	22	12.5 (2.3)	91
Derefinko et al (2009), USA	Cohort	11.8 (1.3)	17 (82)	Mixed	x	≥80, medical files	x	x	x	25	11.4 (1)	89
Flake et al (2007), USA	Cohort	Younger: 5.79 (.87) Older: 8.53 (.86)	80 (76)	WO	C (*n* = 80)	≥80, medical files	WPPSI-III *or* WISC-III	Vocabulary	Below controls	111	Younger: 5.67 (.73) Older: 8.41 (.99)	82
Flory et al (2006), USA	Case-control	8.47 (.85)	49 (79)	WO	x	x	WISC-III	Vocabulary	Below controls	67	8.38 (1)	95
Freer et al (2011), USA	Longitudinal	9 (1.77)	54 (78)	WO	x	x	OWLS	Expressive language	Below controls	101	8.73 (1.76)	91
Hayden et al (2018), USA	Cohort	8.91 (1.23)	23 (69)	WO	x	KBIT-2 Total IQ,109.43 (12.99), Below controls,	OWLS	Expressive language	Below controls	36	9.20 (1.16)	100
Houghton et al. (2007), UK	Cohort	10.2 (1.4)	24 (100)	WO	x	WISC-III-R PIQ, 105.17 (20.86), Comparable to controls	WISC-III-R, Vocabulary, Similarities subtests	Verbal IQ	Comparable to controls	24	10.3 (1.3)	95
Jepsen et al (2024), Denmark	Cohort	9.69 (1.17)	46 (80.4)	WO	x	WISC-IV, 100.68 (16.83), Below controls	CELF-4, ELI, and RLI	Expressive and receptive language	Below controls	40	9.22 (1.41)	100
Kuijper et al (2015), Nether.	Cohort	8;9 (1;7)	37 84)	WO	x	WISC-III-NL FSIQ 95.45 (18.73),Below controls	PPVT; WISC-III-NL	Verbal Ability; Verbal IQ	Below controls	38	9;1 (1;9)	82
Kuijper et al (2017), Nether.	Cohort	8;11 (1;7)	34 (82)	x	x	WISC-III-NL FSIQ 93.09 (17.58) Below controls	PPVT	Verbal ability	Below controls	36	8;11 (1;8)	95,
Lee et al. (2017), South Korea	Cohort	8.0 (0.8)	15 (73)	x	x	≥85, KWISC-III, no comparison collected	KOSECT,	Comprehension	Comparable to controls	15	7.9 (.9)	91
Lorch et al (1999), USA	Cohort	3.4–7.1	27 (81)	WO	PH (*n* = 3); C (*n* = 27)	x	PPVT-R,	Vocabulary	Comparable to controls	52	3.4–7.1	95
Lorch et al (2010), USA	Longitudinal	T1: 7.12 (1.66) T2: 8.91 (1.77)	57 (77)	Nil	C (*n* = 57)	≥80, WPPSI-III *or* WISC-III	WPPSI-III/WISC-III OWLS	Verbal capacity; Listening Comprehension, Oral Expression	All below controls	98	T1: 7.23 (1.59) T2: 8.94 (1.61)	100
Lou and Timler (2008), USA	Cohort	10;9 (1;11)	6 (67)	x	x	WISC-III FSIQ, ADHD only 106 (22); ADHD + LI 94 (12), Comparable to controls	CELF-4	Language status	Below controls	13	10;1 (1;3)	95
Mathers (2006), Australia	Case-control	8–12	11 (82)	WO	x	x	CELF-3, TOPL,	Language ability	Comparable to controls	11	8–12	82
Miniscalco et al (2007), Sweden	Cohort	7;9	8 (88)	x	x	WISC-III FSIQ, 91.4 (8.4), Comparison not conducted	WISC-III (VIQ) NEPSY,	Language ability	All ≥, various performance relative to control/comparison	8	7;9	86
Moonsamy et al (2009), South Africa	Cohort	9;4 (.3), 10;4 (.3), 11.3 (.4)	30 (100)	x	x	IQ WNL (measure unspecified)	x	x	x	n/a	n/a	86
Nilsen et al (2015), Canada	Cohort	10.5 (1.1)	25 (not reported)	Yes	x	x	WIAT-III	Expressive Vocab.	x	29	10.5 (1.1)	91
Papaeliou et al (2015), Greece	Cohort	8.5 (1.4)	25 (68)	Nil	x	WISC-III PIQ, 99 (16.6, Comparable to controls	WISC-II VIQ	Language ability	Vocabulary: Comparable Grammar-comprehension: Below controls. Grammar-production: Comparable to controls	25	9.1 (1.4)	95
Purvis and Tannock (1997), Canada	Cohort	8.7 (1.4)	14 (100)	Mixed	x	WISC-R FSIQADHD only 104.9 (9.4); ADHD + RD 101.6 (12.2), Both comparable to controls,	WISC-R Vocablary; WT, LPT	; Verbal IQ; Expressive vocabulary, semantics	All comparable to controls	14	9.3 (1.9)	82
Redmond (2004), USA	Cohort	6.9 (0.83)	10 (90)	Yes	x	CMMS 99 (8), Comparable to controls,	TOLD-P3	Language ability	Comparable to controls	13	6.5 (.75)	95
Redmond et al. (2011), USA	Cohort	7.9 (.62)	20 (75)	Mixed	x	NNAT-I, 101.15 (10.34), Comparable to controls	CELF-4 Screening Test	Language ability	Comparable to controls	20	7.8 (.53)	91
Redmond et al (2023), USA	Cohort	8.8 (.56)	13 (53.8)	WO	C	NNAT-I 108.54 (11.01), Comparable to controls	NWR, RSR; TEGI	Language ability	Comparison to control not reported	n/a	n/a	91
Redmond et al (2024), USA	Cohort	7.56 (.82)	18 (61)	WO	x	NNAT-I, 108.50 (11.35), Comparable to controls	CELF-5-RS; PPVT-4; TEGI,	Language ability	Comparable to controls	24	7.58 (.94)	100
Renz et al (2003), USA	Cohort	12.02 (2.23)	22 (100)	WO	x	WISC-III Block Design, 9.67 (3.83), Comparable to controls	WISC-III Vocabulary;	Verbal ability	Below controls	44	11.65 (1.01)	86
Rumpf et al (2012), Germany	Cohort	9.9 (1.74)	9 (89)	x	X	WISC-German *or* CFIT-German, Comparable to controls (*M, sd* not provided)	MLU	Language ability	Comparable to controls	11	9.11 (.98)	82
Staikova et al (2013), USA	Cohort	8.62 (1.86)	28 (82)	Nil	x	>80 WISC-IV	CASL, CELF-4	Language ability	All below controls	35	9.08 (1.08)	86
Timler and White (2014), USA	Cohort	6.74 (.66)	32 (84)	Mixed	x	*r* WISC-IV NVIQ 9.52 (3.44); FSIQ 100.33 (14.56), Comparable to controls	CELF-4 CLS	General language ability	Comparable to controls	12	6.76 (1.13)	95
van Lambalgen et al (2008), Nether.	Cohort	7–9	26 (100)	WO	x	x	x	x	x	34	7–9	73
Van Neste et al. (2015), USA	Cohort	8.99 (1.22)	23 (70)	WO	C (*n* = 23)	KBIT-2 Total IQ 109.43 (12.99), Below controls	OWLS	Expressive language	Below controls	35	9.20 (1.18)	91
Zenaro et al (2019), Brazil	Cohort	9.8	20 (70)	Yes	x	x	x	x	x	20	9.8	82
Zentall et al (1988), USA	Cohort	9.17 (1.28)	22 (91)	x	x	WISC *or* Stanford-Binet, 120 (17.2), Comparable to controls	X	x	x	22	8.67 (1.11)	82,
Adult studies												
Coelho et al (2018), Brazil	Cohort	59, 61, 64	3 (33)	x	x	x	Naming, Word Association, Reading, and Writing	Language	Within normal range	2	63, 62	75,
Coelho et al, (2021), Brazil	Cohort	26 (4)	58 (41)	WO	X	WAIS-III, 119 (8)	x	x	x	n/a	n/a	77,
Engelhardt et al (2011), UK	Cohort	23.45 (4.5)	44 (53)	WO	PH (*n* = 6); PI (*n* = 18), C (*n* = 20)	WAIS-III, 111.79 (12.60), Comparable to controls	x	x		31	24.7 × 7 (4.93)	95

*Note*. Study quality was rated as very strong (90–100%), strong (70–89%), adequate (50–69%), or limited (<50%); WO = medication washout at the time of assessment; x = not reported in study; Sub-types: PH = primarily hyperactive/impulsive; PI = primarily inattentive; C = combined.

a As assessed using standardised measures of language ability/verbal capacity (excluding discourse assessments). Where results were presented relative to a neurotypical comparison group, descriptors (e.g., below/comparable/above controls) were used. Due to variability in measures and reporting practices, results are summarised descriptively. BST = The Bus Story Test; CAS = Cognitive Assessment System; CASL = Comprehensive Assessment of Spoken Language; CCC = Children's Communication Checklist; CELF = Clinical Evaluation of Language Fundamentals; CFIT-German = Culture Fair Intelligence Test (German); CMMS = Columbia Mental Maturity Scale; GJ20 = Grammaticality Judgement; KBIT = Kaufman Brief Intelligence Test; KOSECT = Korean Oral Syntax Expression Comprehension Test; KWISC = Wechsler Intelligence Scale for Children (Korean); LPT = Language Processing Test; MLU = mean length of utterance; OWLS = Oral and Written Language Scales; P-FA = Paradise-Fluency Assessment; PPVT = Peabody Picture Vocabulary Test; RE = Recalling Sentences; SR = sentence repetition; SRT = syllable repetition task; NEPSY = Developmental Neuropsychological Assessment; NWR = non-word repetition; TEGI = Test of Early Grammatical Impairment; TNL NLAI = The Test of Narrative Language Narrative Language Ability Index; NNAT-I = Naglieri Non-verbal Ability Test – Individual; TOLP = Test of Language Development Primary; TONI = Test of Nonverbal Intelligence; TONI = Test of Nonverbal Intelligence; TOPL = Test of Pragmatic Language; TROG = Test for Reception of Grammar; VCI = Verbal Comprehension Index; WASI = Wechsler Abbreviated Scale of Intelligence; WIAT = Wechsler Individual Achievement Test; WIPPSI = Wechsler Preschool and Primary Scale of Intelligence; WISC-German = Wechsler Intelligence Scale for Children (German); WRAT = Wide Range Achievement Test; WRMT = Woodcock Reading Mastery Test; WT = The Word Test. (*nb*. R = revised, Roman numerals and numerals indicate editions).

### Participant Characteristics

A total of 2,060 participants were included across studies; and these included 1,047 participants with a confirmed diagnosis of ADHD (*n* = 490 male, *n* children = 942, *n* adults = 105). Thirty-six studies (92%) profiled the discourse abilities of children and adolescents with ADHD (age range four to 16 years). Three studies examined discourse in adults with ADHD (Coelho et al., 2018, 2021; Engelhardt et al., 2011) ranging in age from 18 to 64 years (*n* = 138). Of 39 studies, seven explicitly reported participants’ ADHD subtypes/presentations (i.e., “combined” or “predominantly inattentive” or “predominantly hyperactive/impulsive”) and 23 studies explicitly reported medication status (see Table 2). Participants in 9 of the 39 studies spoke a language other than English, including Brazilian Portuguese (*n* = 1), Dutch (*n* = 2), and Swedish (*n* = 2), as well as Spanish, Korean, Greek, and German (all *n* = 1). Four studies were conducted in settings where English was not the dominant language, but the participants’ languages were not reported. Participant characteristics are outlined in detail in Table 2.

### Discourse Genres

Most studies examined narrative discourse (*n* = 30) using either one or a combination of *retell*^3^ (*n* = 15) or *generation*^4^ tasks (*n* = 17). See Table 3. Other genres (*n* = 11) included *expository (n* = 5), *conversational* (*n* = 4), and *procedural* (*n* = 2) discourse. Three studies analysed discourse samples that combined narrative and non-narrative output, thereby preventing the report of separate findings of narrative and non-narrative categories (Bangert & Finestack, 2020, Mathers, 2006, Timler & White, 2014).

**Table 3.** Discourse Assessment Methods, Outcomes, and Analyses Across Studies.

Author (year)	Discourse assessment		Language level	Group diff reported?	Possible confounder(s) tested (sig?)	Analysis
	Genre	Task (as described)				
Child studies						
Baixauli-Fortea et al. (2018)	NG	Wordless picture book, “Frog goes to dinner”	MiL MaS	~ ✓	Sex (X) Vocabulary (X) Parent education (X)	MANCOVA
Bangert and Finestack (2020)	NR; NG; E; P; C †	ADOS: Narrative generation with (1) picture book stimulus; (2) picture card stimulus; (3) object stimulus (NG). Procedural description “brushing teeth” (P – *note* referred to as “narrative task” in paper). Picture description (E). Structured and unstructured conversation task (C), including personal recount elicitation (NR).	MiL	~	Age (✓) Expressive language (✓) IQ (X)	Wilcoxon signed-rank tests, Kendall’s correlation (possible confounders)
Barkley et al (1983)	C	Play-based interaction	MiL	~	Nil	One-tailed t tests
Bergman and Hallin (2021)	NR	Retell with and without picture support	Mi SuS	✓ ✓	Task (with/without picture support; ~, SuS only)	Two-way repeated ANOVAs
Boo et al. (2022)	C	Structured conversational task (question and answer)	MiL MaL	✓ ✓	IQ (~, MiL only)	Mixed ANCOVAs
Derefinko et al. (2009)	NG	Wordless picture book, “Frog, Where are You?” and “A Boy, a Dog, and a Frog.”	MiL SuS	~ ✓	Nil	One-tailed t tests
Flake et al. (2007)	NR	Retell of televised prompt, “Rugrats”	SuS	✓	Age (✓)	General linear regression
Flory et al. (2006)	NG	Wordless picture book, “Frog, Where are You?” and “A Boy, a Dog, and a Frog.”	MaL SuS	✓ ~	Executive function (X) Vocabulary (~) Phonological processing (~)	One-way ANOVAs Mediation (possible confounders)
Freer et al. (2011)	NG	(1) Generation with no prompt (2) Generation with written/picture cues	MaS	✓	Expressive language (✓) Sex (X)	MANOVAs
Hayden et al. (2018)	NR	Retell of audio-taped stories, “Silly Richard” and “The Brave Knight”	MaS SuS	✓ ✓	Expressive language (X) Sex (X) IQ (X)	one-way ANOVAs
Houghton et al. (2007)	NR	(1) Free retell, non-dialogue video “The Trouble with Mr Bean” (2) Picture-prompted retell, same stimulus	SuS	✓	IQ (X)	Between-groups MANOVA
Jepsen et al. (2024)	NG	Wordless picture book, “Frog, where are you?”	MaS	✓	Sex (X)	Multiple mediation
Kuijper et al. (2015)	NG	Wordless picture books	MaL	✓	Nil	Generalised linear mixed model
Kuijper et al. (2017)	NG	ADOS Storytelling task	MiL MaL	~ ✓	IQ (~, MiL only)	Generalised linear models
Lee et al. (2017)	NR; E	Paradise-Fluency Assessment second Edition – Story retell (NR) and Picture description (E) tasks	MiL	✓	Task (X)	Mixed ANOVA
Lorch et al. (1999)	NR	Retell of televised “Sesame Street” stories	SuS	✓	Age (✓) Y)	General linear regression
Lorch et al. (2010)	NR	Retell of televised prompt, “Rugrats”	MaS SuS	✓ ✓	Age (✓) Expressive language (✓)	MANOVAs
Lou and Timler (2008)	NG	Test of Narrative Language (TNL; Gillam & Pearson, 2004), wordless picture tasks.	SuS	~	Co-occurring language impairment (✓)	Kruskal-Wallis
Mathers (2006)	NG; P†	Animated cartoon generation and narration Description of animation development process	MiL MaS	~ ✓	Nil	Non-parametric t-test
Miniscalco et al. (2007)	NR	The Bus Story (Renfrew, 1995; 1997)	MiL SuS	✓ ✓	Co-occurring Language impairment (✓)	Fisher’s exact test
Moonsamy et al. (2009)	NG; NR†	(1) Retell of being “hurt or scared” (2) Generation using picture sequence cards	MaL	✓	Nil	Descriptive
Nilsen et al. (2015)	E	Picture description to naïve listener	MaL MaS	✓ ✓	Working memory (✓) Executive function (X)	Regression
Papaeliou et al .(2015)	NR	Recall task, “The Father, His Son, and Their Donkey”	SuS	✓	Working memory (✓) Vocabulary (X)	MANOVA
Purvis and Tannock (1997)	NR	Recall task, “The Father, His Son, and Their Donkey”	MiL MaL MaS	~ ✓ ✓	Age (X) IQ (X)	ANOVA
Redmond (2004)	NG; C	Conversation and structured narrative recount during free play	MiL	~	Nil	ANOVA
Redmond et al. (2011)	NG	TNL (Gillam & Pearson, 2004)	Combined	✓	Nil	ANOVA
Redmond et al. (2023)	NG	Test of Narrative Language-Second Edition (TNL-2: Gillam & Pearson, 2017)	Combined	n/a	Nil	Paired-samples t tests
Redmond et al. (2024)	NG	TNL-2 (Gillam & Pearson, 2017)	Combined	Nil	Co-occurring language impairment (✓) Executive function (X)	ANOVA
Renz et al. (2003)	NG	Wordless picture book, “Frog, where are you?”	MaL MaS SuS	✓ ✓ ~	IQ (X)	MANOVA
Rumpf et al. (2012)	NG	ADOS Storytelling task	MiL MaL	~ ~	Nil	Kruskal–Wallis
Staikova et al. (2013)	NG	Narrative Assessment Profile (Bliss et al., 1998)	Combined	✓	General language ability (n/r)	MANCOVA
Timler and White (2014)	NG; NR; C†	TNL (Gillam & Pearson, 2004; NG) Conversation sample (C) including narrative retell elicitation.	Combined	✓	Co-occurring language impairment (✓)	ANOVAs
van Lambalgen et al. (2008)	NG	Wordless picture book, “Frog, where are you?”	MiL	~	Nil	Mann-Whitney U
Van Neste et al. (2015)	NR	Retell of televised episodes, “Growing Pains”	MaS	✓	IQ (X) Expressive language (X) Mother’s education (X)	ANCOVAs, Mixed ANOVAs
Zenaro et al. (2019)	NR	Wordless picture book, “Frog, where are you?”	MaS SuS	✓ ~	Nil	Likelihood ratios
Zentall et al. (1988)	NR; NG†	Four study-specific tasks: free narrative generation, word-prompted narratives, picture-prompted narratives, story retell.	MiL MaL MaS	✓ ✓ ✓	Nil	ANOVA
Adult studies						
Coelho et al. (2018)	NG	Wordless picture book, “Frog, where are you?”	MiL MaS SuS	✓ ✓ ✓	Nil	Speech Graphs Analysis
Coelho et al. (2021)	NG	Wordless picture book, “Frog, where are you?”	MiL MaL MaS	✓ ✓ ✓	IQ (X) Education (X) Depression (X) Anxiety (X)	Correlation
Engelhardt et al. (2011)	E	Visually guided expository description task	MiL SuS	✓	Age (✓) IQ (X) Reading ability (X)	MANOVA Correlation (possible confounders)

*Note*. G = Narrative generation; NR = Narrative retell; E = Exposition; P= Procedure; C = Conversation. † Genre samples combined for analysis. MiL = micro-linguistic; MaL = macro-linguistic; MaS = macro-structural; SuS = super-structural. ✓ = Yes; X = No; ~ = mixed results reported; n/r = not reported. M/ANOVA = multivariate/analysis of variance; MANCOVA = multivariate analysis of covariance.

### Discourse Assessment Tools

Five studies utilised standardised assessments to elicit discourse samples. These tools included the Autism Diagnostic Observation Schedule-2 (Lord et al., 2000; *n* = 3) and the Test of Narrative Language (Gillam & Pearson, 2004; *n* = 5), as well as the Bus Story Test (Renfrew, 1995), the Detroit Test of Learning Abilities Story Construction subtest (Hammill, 1991), and the narrative memory subtest in NEPSY (“A Developmental Neuropsychological Assessment”; Korkman et al., 1998; each *n* = 1). The authors of the remaining 33 studies utilised non-standardised assessments such as free play, wordless storybooks, picture descriptions, or televised stimuli retell tasks.

### Discourse Characteristics Across Levels of Analysis

The results of the included studies are summarised by each level of analysis. Where possible, the results are presented and grouped under narrative and non-narrative discourse. Due to the small number of studies of adults with ADHD that met inclusion criteria, the results of studies of children with ADHD are presented first, followed by a separate section on adults with the disorder. Regarding the four analysis levels, most studies analysed superstructural (*n* = 23) or micro-linguistic language features (*n* = 23); followed by macro-structural (*n* = 13), then macro-linguistic (*n* = 12).

Micro-linguistic language features examined structures within a sentence. These included sentence length (*n* = 9), productivity (total words produced *n* = 8, total utterances *n* = 6), filled pauses (use of “um” or “like,” *n* = 8), lexical diversity (*n* = 6), grammatical complexity (*n* = 6), speech rate (*n* = 3), verb and noun types (*n* = 2), utterance revisions (*n* = 2), dysfluencies (e.g., repeated word[s]), complexity (*n* = 5), and word-level errors (e.g., word substitutions, *n* = 3).

Fewer studies analysed macro-linguistic (*n* = 12) and macro-structural (*n* = 13) discourse features, predominantly in children. Macro-linguistic measures included cohesive adequacy or ambiguity (*n* = 10) and the total number of cohesive devices such as pronouns (*n* = 4) and conjunctions (*n* = 4).

Macro-structural measures included indices of global coherence (*n* = 6), informativeness or redundancy of information (*n* = 8), and efficiency of information transfer (*n* = 2). Super-structural features included the number core narrative components (*n* = 15), narrative elements retold (*n* = 6), general measures of “overall” structure (*n* = 3), atypical topic changes (*n* = 2), and errors in narrative sequencing (*n* = 2).

### Narrative Discourse in Children With ADHD

#### Micro-Linguistic Features

In children with ADHD, researchers reported fewer words, reduced sentence length and fewer important details (i.e., atypical brevity) than neurotypical peers in response to narrative *generation* tasks (Rumpf et al., 2012; Zentall, 1988). Studies also reported reduced sentence complexity (Kuijper et al., 2017; Rumpf et al., 2012; van Lambalgen et al., 2008) and higher frequency of grammatical errors (Kuijper et al., 2017; van Lambalgen et al., 2008), and more dysfluencies (e.g., word repetitions and filled pauses; Bangert & Finestack, 2020; Kuijper et al., 2017; Lee et al., 2017) in children with ADHD. In contrast, Zentall (1988) detected significantly more dysfluency errors in *neurotypical* compared to hyperactive children. Regarding narrative *retell*, authors reported in children with ADHD more word-level and grammatical errors (Bergman & Hallin, 2021; Purvis & Tannock, 1997), and reduced sentence complexity (Bergman & Hallin, 2021; Miniscalco et al., 2007). However, nine studies that examined micro-linguistic features reported within-study mixed results: that is, both significant and non-significant differences between children with and without ADHD were found within each study at this level of analysis (Baixauli-Fortea et al., 2018; Bangert & Finestack, 2020; Barkley et al., 1983; Derefinko et al., 2009; Kuijper et al., 2017; Mathers, 2006; Redmond, 2004; Rumpf et al., 2012; van Lambalgen et al., 2008).

#### Macro-Linguistic Features

Regarding narrative *generation* tasks, four studies reported significantly reduced cohesive adequacy and frequency in children with ADHD (Flory et al., 2006; Kuijper et al., 2015; Renz et al., 2003). However, one study reported no differences in quantity of cohesive devices in affected cases (Rumpf et al., 2012). Two studies combined narrative *generation* and *retell* tasks, and found fewer pronouns and conjunctions produced in child with ADHD (Moonsamy et al 2009; Zentall, 1988). Similarly, Purvis and Tannock (1997) reported more ambiguous referencing (e.g., the use of “he” without specifying the referent, such as “John”) in narrative *retell* in affected children.

#### Macro-Structural Features

In children with ADHD, researchers consistently found the presence of inefficient and/or redundant information (i.e. irrelevant or tangential content) and less coherent output (i.e., reduced flow of ideas between sentences) in narrative *generation* (Flory et al., 2006; Freer et al., 2011; Mathers, 2006; Renz et al., 2003; Staikova et al., 2013; Zenaro et al., 2019; Zentall, 1988) and narrative *retell* samples (Hayden et al., 2018; Jepsen et al., 2024; Lorch et al., 2010; Purvis & Tannock, 1997; Redmond et al., 2011;Van Neste et al., 2015; Zentall, 1988). For example, inefficient or tangential content might be reflected in an utterance such as: “*we went to the park and saw ducks. I had chicken nuggets for lunch yesterday. Then we fed the ducks*,” where the second sentence introduces irrelevant information that disrupts the narrative flow. Reduced coherence, on the other hand, may be evident in an example such as: “*we went to the park. There were swings. It was fun. Then home*,” where the ideas are presented as disjointed fragments with minimal connective language, resulting in a lack of clear relationships between events. One study (Baixauli-Fortea et al., 2018) reported more redundant content in narrative *generation* samples of children with co-occurring ADHD + ASD compared to children with and without ADHD.

#### Super-Structural Features

Researchers reported that participants with ADHD produced significantly fewer essential narrative elements (e.g., main events and endings) and more errors in narrative structure (e.g., producing the main event before identifying key people or characters) in response to narrative *generation* tasks (Derefinko et al., 2009; Flory et al., 2006; Luo & Timler, 2008; Redmond et al., 2011; Renz et al., 2003; Staikova et al., 2013; Timler & White, 2014; Zenaro et al., 2018) and narrative *retell* tasks (Bergman & Hallin, 2021; Flake et al., 2007; Hayden et al., 2018; Houghton et al., 2007; Lorch et al., 1999, Papaeliou et al., 2015; Zentall, 1988). In contrast, two studies reported no differences in narrative structure between children with and without ADHD (Luo & Timler, 2008; Miniscalco et al., 2007).

### Omnibus Narrative Score

Five studies characterised narrative output using a total or “overall” score to represent discourse skill (Redmond et al., 2011; Staikova et al., 2013; Timler & White, 2014). The term “omnibus narrative score” is taken here to mean a single, composite score intended to reflect overall narrative ability. This score is derived by combining multiple metrics that assess discrete narrative language skills, across micro-linguistic to super-structural levels, into one index. As a result, it is not possible to disaggregate the contribution of individual language skills within the overall score. Three studies (Redmond et al., 2011, 2024; Timler & White, 2014) used the Narrative Ability Index (NAI) generated from the Test of Narrative Language (Gillam & Pearson, 2004). Timler and White (2014) reported significantly lower NAI scores in children with ADHD compared to neurotypical peers, all aged 5 to 8 years. Conversely, Redmond et al. (2011, 2024) did not report significant differences in the NAI scores in children with ADHD aged 7 to 8 years. Redmond et al. (2023) also did not report significant differences between children with and without ADHD on the Test of Narrative Language using the specific Narrative Production score. Staikova et al. (2013) used an overall score generated by the Narrative Assessment Profile (Bliss et al., 1998), which reflects macro-linguistic to super-structural features. The authors reported lower Narrative Assessment Profile scores in children with ADHD compared to neurotypical controls. Due to the nature of omnibus scores, it is not possible to determine whether specific language features (i.e., micro-linguistic to super-structural features) were affected by ADHD. Overall, patterns across studies showed that ADHD is associated with narratives of atypical length (verbosity and/or brevity),reduced syntactic and grammatical complexity and poorer coherence. Additionally, stories told by speakers with ADHD tended to include redundant information and/or omit critical narrative components.

### Non-Narrative Discourse in Children With ADHD

#### Micro-Linguistic Features

Six studies examined micro-linguistic features of non-narrative discourse samples, five in children and one in adults. In children, studies reported significantly more dysfluent (Lee et al., 2017; Redmond et al., 2004), as well as longer and more verbose *conversational* output for those with ADHD (Barkley et al., 1983; Boo et al., 2022). One study (Mathers, 2006) reported a greater number of incomplete sentences within samples that were a combination of procedural, narrative generation, and narrative retell output (Mathers, 2006). In contrast, Timler and White (2014) reported no significant differences between children with and without ADHD on micro-linguistic features in narrative retell *and* expository discourse.

#### Macro-Linguistic Features

In children, one study reported fewer pronouns in *conversation* for ADHD participants (Boo et al., 2022); and one other found more ambiguous referencing in the *expository discourse* when required to describe an object to an unfamiliar listener (Nilsen et al., 2015).

#### Macro-Structural Features

Nilsen et al. (2015) was the only study to explicitly analyse macro-structural features of non-narrative discourse produced by children with ADHD. The authors reported significantly reduced informativeness in *expository* discourse in children with ADHD relative to typically developing peers. Mathers (2006) examined macro-structural features in combined *procedural* and narrative discourse produced by children with ADHD; and found more redundant and tangential information compared to neurotypical controls.

#### Super-Structural Features

Only one study examined super-structural features in non-narrative discourse mixed with narrative analysis in children with ADHD. Mathers (2006) reported fewer stereotypical discourse components (e.g., ending or concluding remarks) in samples that included combination of *procedural*, narrative retell, and narrative generation discourse relative to neurotypical peers.

### Narrative Discourse in Adults With ADHD

Two studies profiled micro-linguistic narrative features in adults with and without ADHD. Notably, these studies reported significantly longer and more tangential output (i.e., verbosity; Coelho et al., 2018, 2021), differing from the results of child studies. At the macro-linguistic level, one study (Coelho et al., 2021) of adults with ADHD reported significantly more ambiguous referencing and a reduced quantity of cohesive devices in response to a narrative *generation* task relative to those without the disorder (thereby reflecting the results of child studies). At the macro-structural level, two studies (Coelho et al., 2018, 2021) reported reduced coherence and redundant information in response to narrative *generation* tasks. Finally, at the super-structural level, one study (Coelho et al., 2018) reported significantly more errors in narrative sequencing and fewer essential elements were found (e.g., main events and ending) in a narrative generation task, corroborating those of children.

### Non-Narrative Discourse in Adults With ADHD

One study (Engelhardt et al., 2011) reported significantly longer and more dysfluent (e.g., unfilled pauses and word repetitions) expository discourse in adults with ADHD relative to controls (Engelhardt et al., 2011). The same study examined super-structure of expository discourse and reported that adults with ADHD structured their ideas in a more basic and simpler fashion than their neurotypical peers, despite the longer length (Engelhardt et al., 2011). However, this characteristic was only observed in adults with “combined subtype” ADHD and not in participants with “predominantly hyperactive-impulsive subtype” or “predominantly inattentive subtype” (Engelhardt et al., 2011). No studies examined macro-linguistic or macro-structural features of non-narrative discourse in adults with ADHD.

Generally, a similar pattern of results was observed for non-narrative and narrative discourse associated with ADHD, showing that discourse differences associated with the disorder may go beyond the narrative genre. That is, expository, conversational, descriptive, and procedural data tended to elicit language samples that differed in length, and in being less complex, less informative, and more poorly structured in speakers with ADHD when compared to neurotypical participants.

### Possible Confounding Factors

As shown in Table 3, most studies (*n* = 26) examined one or more potential confounding variables in the relationship between ADHD and discourse output, though the inclusion, measurement, and significance of these variables varied widely. The most frequently tested confounders were IQ (*n* = 10), age (*n* = 6), and expressive language ability (as a continuous variable; *n* = 5), followed by co-occurring language disorder (*n* = 4), and executive function, vocabulary, education, and sex (each *n* = 3). Working memory was examined in two studies, while internalising symptoms (anxiety and depression) and picture support were each tested in one. Age and co-occurring language disorder were the most consistently significant, with older participants and those without a diagnosed language disorder typically demonstrating less disrupted discourse across studies (e.g., Lorch et al., 1999; 2010; Luo & Timler, 2010; Miniscalco et al., 2007). In contrast, findings related to IQ (e.g., Kuijper et al., 2017; Renz et al., 2003), expressive language (as a continuous variable; Lorch et al., 2010; Hayden et al., 2018), vocabulary (e.g., Flory et al., 2006), and executive function (e.g., Nilsen et al., 2015) were inconsistent.

### Additional Findings

While not the focus of this review, we noted that three of the reviewed studies reported on the effect of medication on discourse features. Two studies (Derefinko et al., 2009; Redmond et al., 2023) examined the effect of stimulant medication on narrative discourse in children with ADHD and one study (Barkley et al., 1983); and tested the influence of medication on micro-linguistic features of conversational discourse. While Redmond et al. (2023) reported no significant effect of medication on narrative production, Derefinko et al. (2009) reported positive effects on narrative organisation. Barkley et al. (1983) found methylphenidate significantly reducing atypical verbosity in conversational discourse, yet the medication yielded no beneficial effect on grammatical complexity and sentence structure.

## Discussion

This systematic review applied a novel four-level analytic framework to synthesise published literature on *narrative* and *non-narrative* discourse capabilities in speakers with ADHD. To our knowledge, it is the most comprehensive review on this topic to date.

Overall, there are three key findings of this review. First, most literature has focussed on narrative discourse in ADHD; only nine studies evaluated non-narrative genres (e.g., conversation and exposition). Only three studies have examined discourse-level language in *adults* with the disorder (two on narrative, one on expository discourse skills). Second, the existing body of literature indicates that ADHD can affect discourse-level language skills across narrative and non-narrative tasks. Specifically, patterns suggest that discourse output in ADHD is characterised by atypical length and detail as well as less efficient and disorganised output relative to neurotypical speakers. Third, our review indicates that study findings appeared to vary most consistently according to age, genre, and task. For example, child participants with ADHD tended to under-produce output (i.e., brevity) in response to structured task situations (e.g., standardised narrative assessments), but provided more verbose and digressive responses (i.e., excessive talking) in unstructured tasks (e.g., conversation). In contrast, though limited, evidence suggests that adults with ADHD may produce longer and more tangential output irrespective of genre and task type. Generally, the relevant literature includes studies of high quality, with clear descriptions of study methodology and detailed results. Our results indicated that *both* children and adults with ADHD may produce discourse output that is discernibly different from neurotypical peers.

Interestingly, the “excessive talking” core diagnostic symptom of ADHD, as described in the DSM-5-TR (APA, 2022), was inconsistently reported by studies included in this review. Most studies of children with ADHD reported *shorter* and less informative discourse output compared to their peers in response to prompted and structured tasks (typically narratives), which is consistent with previous reviews (Carruthers et al., 2021; Jepsen et al., 2022). In contrast, excessive and incoherent output among *children* was only reported in a few studies; this tended to be in response to less structured probes, such as conversations. Similarly, though informed by only a few studies, it appears that the profile of discourse in *adults* with ADHD is also more in keeping with the DSM criterion. Adult participants were observed to be consistently verbose, including too many or redundant details in both narrative and non-narrative tasks, thus differing from children.

This finding suggests that individuals with ADHD may either under-produce or over-produce discourse depending on age, task type, and communicative context. Although only a few studies have examined adults with ADHD, their findings suggest reduced responsiveness to the scaffolding provided by structured tasks. This pattern is consistent with differences in the cognitive and linguistic demands of discourse contexts. While narrative tasks are typically monologic in research and clinical settings, they nonetheless involve subtle co-construction with a listener and require sensitivity to audience cues and contextual relevance (Spencer & Petersen, 2020); despite these demands, their structured format can support discourse planning. By contrast, unstructured interactions (e.g., free conversation) require greater flexibility, topic management, and conversational repair, thereby possibly imposing heavier executive-control demands (Hill et al., 2018). Additionally, adults may adopt compensatory strategies, such as over-talking to maintain engagement or mask disorganisation, that may persist even when structure is present (Kysow et al., 2017). Developmental factors may also contribute. Adults may be able to sustain longer output, making verbosity and tangentiality more likely when regulation is poor, whereas children’s developing planning and working-memory resources could constrain output when prompted in structured tasks. Indeed, convergent evidence shows increasing syntactic complexity and content from childhood to adulthood and stronger scaffolding effects of narrative tasks within childhood (Frizelle et al., 2018; Lindgren, 2023). In addition, adults may interpret structured elicitation as evaluative and increase talkativeness accordingly, consistent with self-presentation motives and audience-design adaptations in adult discourse production (Burd, 2013; Horton & Spieler, 2007). Together, these factors may help explain adults’ relative insensitivity to contextual scaffolding compared with children with ADHD. A systematic examination of how task structure and support shape discourse in children versus adults with ADHD remains a priority for future research.

Difficulties with the “basic” components of language structure (e.g., grammar and sentence structure) in ADHD have been previously reported (Korrel et al., 2017). Jepsen et al. (2022) suggested that micro-linguistic level errors produced by children with ADHD are indicative of poorer knowledge and skills in vocabulary, morphology (grammar), and syntax. We extend this perspective and suggest that, in the absence of clinical language disorders (as was the case for most participants), observed difficulties with the “building blocks” of language during discourse production (i.e., vocabulary, grammar, and syntax) also reflect the broader profile of ADHD. Here, we suggest that variability in attentional resources, working memory, and executive function may lead to differences in how individuals with ADHD apply their knowledge of vocabulary, grammar, and syntax to discourse-level communication. For instance, difficulty adequately attending to and planning discourse output may result in increased dysfluencies, pauses, and grammatical errors, even in the absence of impaired knowledge of discrete linguistic components in children and adults with ADHD (Coelho et al., 2021; Kuijper et al., 2017).

Despite this, some studies reported significantly poorer general language ability in individuals with ADHD compared to controls, based on standardised language assessments (e.g., Bergman & Hallin, 2021; Hayden et al., 2018) or verbal IQ measures used as proxies for language ability (e.g., Baixauli-Fortea et al., 2018; Flake et al., 2007; Flory et al., 2006). The co-occurrence of poor discourse and lower performance on these measures supports the possibility that discourse-level difficulties attributed to attentional or executive deficits may be further compounded by underlying structural language impairments. This interpretation is reinforced by findings from three studies (Lou & Timler, 2008; Redmond et al., 2024; Timler & White, 2014), which showed that children with both ADHD and a diagnosed clinical language disorder performed more poorly on discourse-level language tasks than those with ADHD alone.

It is important to note that several included studies provided limited evidence that general language ability, or “verbal capacity,” functions as a consistent confounder of discourse-level language competence in ADHD (e.g., Freer et al., 2011; Hayden et al., 2018). Across studies, the measures used to assess general language ability, often employed as proxies for underlying language capacity rather than formal diagnoses of co-occurring language disorder, may not accurately reflect an individual’s true structural language profile. For example, the WISC Vocabulary subtest focusses on a narrow domain (i.e., receptive vocabulary), and performance is influenced by cognitive and sociocultural factors (e.g., working memory, cultural, and educational background; Attout et al., 2020; Weiss & Saklofske, 2020). Similarly, the CELF Recalling Sentences subtest (heavily reliant on auditory working memory) assesses discrete and cognitively demanding skills (Calder et al., 2023). Indeed, studies have encouraged caution when using these and similar measures as proxies for language ability, including within neurodivergent populations (Calder et al., 2023; de Oliveira et al., 2020).

Given the close interplay between language and cognition, there is a risk that these standardised tools conflate cognitive profiles in ADHD with true underlying language capacity. In contrast, broader composite indices, such as the CELF Expressive and Receptive Language Indices (e.g., as used in Jepsen et al., 2024), may provide a more comprehensive and accurate estimate of structural language ability beyond narrow, domain-specific tasks. The evidence base would benefit from a more consistent and thorough assessment of underlying structural language abilities to better understand how these profiles interact with the cognitive characteristics of ADHD in shaping discourse-level language performance. Additionally, no studies in adults with ADHD examined language ability or the presence of co-occurring language impairment as potential confounders, limiting our understanding of their contribution to communicative outcomes in this population.

Our review also highlighted that children *and* adults with ADHD demonstrated difficulty in using cohesive devices to organise and logically connect ideas during narrative and non-narrative tasks. Our finding suggests that ADHD may disrupt retrieval of specific contextual information, such as names of people/characters or settings when needed to recount a factual or emotional event. These problems likely reflect a difficulty in gauging listeners’ prior knowledge and suppressing one’s perspective to provide context not explicitly shared between communication partners (Pineda-Alhucema et al., 2018; Tatar & Cansız 2022). This finding indicates that children and adults with ADHD who *do not* have a co-occurring language disorder may also struggle to provide key contextual information to communication partners. This lack of context can have a considerable impact on communication success with key communication partners, including mental health professionals, educators, and peers.

Studies reported that children and adults with ADHD produced more redundant and superfluous information and less coherent discourse in both narrative and non-narrative genres. Their output may be overly detailed with an inefficient rate of information transfer, or they may be too brief, failing to convey enough information to adequately inform the listener (Coelho et al., 2018, 2021). One study identified that adults with ADHD exhibit a disorganised structure when producing expository discourse (Nilsen, 2015). These characteristics may also be related to inattention and impulsivity, which contribute to a failure to inhibit less relevant information and produce critical information to communicate efficiently (Kuijper et al., 2015, 2017; Nilsen et al., 2015). Extending the findings of Jepsen et al. (2022), our results indicate both children and adults with ADHD have difficulties in including and sequencing essential discourse components across both narrative and non-narrative genres.

In summary, discourse-level language in ADHD is affected at the levels of structure and content across narrative and non-narrative contexts in children and adults. However, the nature and severity of these difficulties may vary within and across genres, depending on the level of structure and scaffolding, which in turn affects cognitive demands and influences performance. Similarly, discourse-level language competence may be influenced by underlying language capacity, particularly in the context of co-occurring clinical language disorders. Our review indicates age group-specific patterns in discourse output may be present. Children with ADHD may generally produce shorter, less informative output in structured narrative tasks yet become more verbose and digressive in unstructured conversation, whereas the limited adult literature indicates a more consistent tendency towards verbosity and tangentiality across task types. These apparent differences should be interpreted cautiously, as they may reflect methodological factors (e.g., task design and sample characteristics) rather than developmental change. Notably, we observed a marked paucity of research focussed on adolescents, limiting our ability to conclude how ADHD affects discourse during this critical developmental stage. Despite our broad inclusion criteria (in age and discourse genre) this gap echoes the observation made by Jepsen et al. (2022) who reviewed few studies of adolescent participants. Indeed, this observation is unexpected, given that adolescence is characterised by rapid development of cognitive maturation, particularly in executive and attentional processes (Hobbiss & Lavie, 2024; Shroff et al., 2024), as well as increasing demands for sophisticated language use in both academic and social contexts (Hill, Claessen, et al., 2021; Hill, Whitworth, et al., 2021). Research in this critical developmental window represents a fertile arena to explore the interaction between language and attentional processes between childhood and adulthood as proficient discourse production during this period is closely tied to social success, identity development, and academic participation (Hill, Claessen, et al., 2021). These findings provide an impetus for the routine assessment of narrative and non-narrative discourse in ADHD; particularly that which characterises discourse output using a comprehensive set of structure and content measures in addition to standardised measures of language ability.

### Limitations and Future Directions

The overall quality of the included studies was strong; however, studies varied in the detail with which authors described the recruitment and selection of participants with ADHD. The evidence would be further strengthened by more detailed delineation of participant sampling, diagnostic processes, and ADHD subtyping to facilitate more in-depth synthesis and interpretation of results. Secondly, we included only peer-reviewed studies published in English. There may be relevant research published in non-English languages, informative on the topic. The extent to which discourse structural analyses in different languages can be combined remains untested at this stage; we, therefore, consider it prudent to confine our review to one single language. Finally, while not a focus of this review, we noted that two studies examined the impact of medication on discourse output in ADHD. We believe a synthesis of the effect of pharmacological intervention on language in ADHD warranted is a valuable direction for a future study.

Beyond the increased prevalence of formal language disorder in individuals with ADHD, the condition can be linked to observable differences in discourse production. The nature of an individual’s discourse-level language features may not be entirely reflected by formal diagnostic categories (i.e., excessive talking). Difficulties with discourse can adversely affect daily functioning within social, educational, and occupational contexts. Given the high prevalence of ADHD in clinical populations (32% in paediatric and 21% in adult populations, respectively; Johnson et al., 2025), a substantial number of individuals seeking support are likely to experience variability in discourse-level language. However, discourse skills across genres are not routinely assessed in clinical settings.

Despite our broad inclusion criteria, most included studies examined narrative skills. We believe this finding reflects a relatively narrow focus of the current literature. This gap highlights the need for researchers to employ a broader focus on a range of discourse genres that are important for daily communication (e.g., persuasive and exposition). Secondly, the available literature is dominated by studies of childhood ADHD and male participants. The marked paucity of studies involving adults with ADHD weakens the evidence base and constrains our ability to draw firm conclusions about the impact of ADHD on discourse in adulthood, or how this may differ from patterns observed in children. Similarly, there is a surprising lack of research focussed on adolescents, despite this being a developmental period marked by significant cognitive changes and increasing demands for sophisticated language use in academic and social contexts. This gap limits our understanding of how ADHD affects discourse during a stage when effective communication is critical for identity formation, peer relationships, and educational success. Further, although meta-analyses have reported mixed or negligible effects of sex on language processing in the general population (e.g., Marini, 2022; Sato, 2020), sex may interact with ADHD to produce differential discourse profiles. Future research should explore these potential interactions, as well as other confounding variables such as underlying language ability and IQ, using consistent and comprehensive measurement, to better understand individual variability in discourse competence. Additionally, researchers should conduct thorough investigations into the micro-linguistic to super-structural analyses of discourse features associated with ADHD in children, adolescents, *and* adults across narrative and non-narrative genres (Hill, Claessen, et al., 2021; Whitworth et al., 2015). In doing so, it may be helpful to move beyond single-speaker narrative tasks and consider how discourse is often co-constructed in real-life interactions. Indeed, an individual must utilise different discourse genres to fully participate in mental health services given clinical dialogue is often complex. For instance, a patient needs to give a psychiatric history (narrative) or an account of facts (expository), describe a process (procedural), negotiate treatment options (persuasion), and engage with psychological therapy (conversation; Hobson et al., 2022). In-depth investigations of discourse in ADHD are required to understand the functional impact of the disorder on effective communication within social, academic, and community environments across the lifespan. Most of the included studies were conducted in English-speaking contexts, with limited representation of other languages. This over-representation of English-speaking participants may constrain the generalisability of findings, as discourse structures and language use vary cross-linguistically (Taylor & del Fante, 2020), which may influence observed discourse profiles in individuals with ADHD. Given the limited number of studies involving non-English-speaking participants, it is difficult to identify consistent patterns when comparing these studies to those conducted in English-speaking contexts. Consequently, future research should examine discourse across diverse linguistic and cultural contexts. This is critical to ensuring that research reflects the heterogeneity of clinical populations. Finally, as described earlier, a meta-analysis of the included studies was not undertaken due to the aim of this review to provide a descriptive summary of extant literature and the substantial methodological heterogeneity, including variation in discourse elicitation tasks (across narrative and non-narrative genres), outcome measures, sample characteristics, and reporting practices (Higgins et al., 2022). A meta-analysis specific to narrative abilities only among children with ADHD has been previously reported by Jepsen et al (2022).

## Conclusions

To our knowledge, this review is the first to provide a comprehensive synthesis of the extant literature on discourse capabilities associated with ADHD. We reviewed studies of children and adults with the disorder, studies of narrative and non-narrative genres, and interpreted findings utilising a four-level framework of micro-linguistic to super-structural discourse features. Overall, studies indicated that ADHD could affect discourse production, particularly productivity, complexity, informativeness, and structure of children and adults with the condition. The extent and nature of ADHD-related discourse characteristics appear to vary according to age and genre under investigation. Our findings are relevant for clinical practice and further research.

Our submission in this review is that all disciplines involved in supporting children and adults with ADHD should be aware of the impact of ADHD on discourse-level language across daily contexts. Routine assessment of a range of discourse genres should be a practice standard. Further, ongoing investigation of methods to scaffold individuals with ADHD to express themselves successfully within clinical, social, and academic contexts is a research imperative.

## Supplemental Material

sj-docx-1-jad-10.1177_10870547251389329 – Supplemental material for Narrative and Non-Narrative Discourse Skills in ADHD Across the Lifespan: A Systematic Review of the Literature Supplemental material, sj-docx-1-jad-10.1177_10870547251389329 for Narrative and Non-Narrative Discourse Skills in ADHD Across the Lifespan: A Systematic Review of the Literature by Elizabeth Hill, Robert Wells and Wai Chen in Journal of Attention Disorders

sj-docx-2-jad-10.1177_10870547251389329 – Supplemental material for Narrative and Non-Narrative Discourse Skills in ADHD Across the Lifespan: A Systematic Review of the Literature Supplemental material, sj-docx-2-jad-10.1177_10870547251389329 for Narrative and Non-Narrative Discourse Skills in ADHD Across the Lifespan: A Systematic Review of the Literature by Elizabeth Hill, Robert Wells and Wai Chen in Journal of Attention Disorders

sj-docx-3-jad-10.1177_10870547251389329 – Supplemental material for Narrative and Non-Narrative Discourse Skills in ADHD Across the Lifespan: A Systematic Review of the Literature Supplemental material, sj-docx-3-jad-10.1177_10870547251389329 for Narrative and Non-Narrative Discourse Skills in ADHD Across the Lifespan: A Systematic Review of the Literature by Elizabeth Hill, Robert Wells and Wai Chen in Journal of Attention Disorders

## Notes

1. Narrative tasks involve factual or fictional storytelling by sequencing events in a causal and/or temporal order.

2. A more detailed description of each level of analysis has been provided in Supplemental Materials.

3. Narrative *retell* tasks require the participant to hear or view a story and retell the narrative after a set period of time.

4. Narrative *generation* tasks require the participant to generate their own narrative, typically prompted by a visual or verbal stimulus (e.g., “Tell me a story based on this image”).

## References

[bibr1-10870547251389329] American Psychiatric Association. (2022). Diagnostic and statistical manual of mental disorders (5th ed., text rev.). American Psychiatric Publishing.

[bibr2-10870547251389329] Attout L. Grégoire C. Majerus S. (2020). How robust is the link between working memory for serial order and lexical skills in children? Cognitive Development, 53, Article 100854. 10.1016/j.cogdev.2020.100854

[bibr3-10870547251389329] *Baixauli-Fortea I. Forner C. B. Colomer C. Casas A. M. Miranda B. R. (2018). Communicative skills in Spanish children with autism spectrum disorder and children with attention deficit hyperactivity disorder: Analysis through parents’ perceptions and narrative production. Research in Autism Spectrum Disorders, 50, 22–31. 10.1016/j.rasd.2018.02.006

[bibr4-10870547251389329] *Bangert K. J. Finestack L. H. (2020). Linguistic maze production by children and adolescents with attention-deficit/hyperactivity disorder. Journal of Speech, Language, and Hearing Research, 63(1), 274–285. 10.1044/2019_JSLHR-19-00187 PMC7213479 31944883

[bibr5-10870547251389329] *Barkley R. A. Cunningham C. E. Karlsson J. (1983). The speech of hyperactive children and their mothers: Comparison with normal children and stimulant drug effects. Journal of Learning Disabilities, 16(2), 105–110. 10.1177/002221948301600209 6842069

[bibr6-10870547251389329] Barkley R. A. Fischer M. Smallish L. Fletcher K. (2006). Young adult outcome of hyperactive children: Adaptive functioning in major life activities. Journal of the American Academy of Child & Adolescent Psychiatry, 45(2), 192–202. 10.1097/01.chi.0000189134.97436.e2 16429090

[bibr7-10870547251389329] *Bergman A. Hallin A. E. (2021). The effect of picture support on narrative retells in Swedish adolescents with ADHD. Clinical Linguistics & Phonetics, 35(7), 690–705. 10.1080/02699206.2020.1825816 32985272

[bibr8-10870547251389329] Bliss L. S. McCabe A. Miranda A. E. (1998). Narrative assessment profile: Discourse analysis for school-age children. Journal of Communication Disorders, 31(4), 347–363. 10.1016/s0021-9924(98)00009-4 9697044

[bibr9-10870547251389329] *Boo C. Alpers-Leon N. McIntyre N. Mundy P. Naigles L. (2022). Conversation during a virtual reality task reveals new structural language profiles of children with ASD, ADHD, and comorbid symptoms of both. Journal of Autism and Developmental Disorders, 52, 1–14. 10.1007/s10803-021-05175-6 34244916

[bibr10-10870547251389329] Botting N. (2002). Narrative as a tool for the assessment of linguistic and pragmatic impairments. Child Language Teaching and Therapy, 18(1), 1–21. 10.1191/0265659002ct224

[bibr11-10870547251389329] Bryant L. Spencer E. Ferguson A. (2017). Clinical use of linguistic discourse analysis for the assessment of language in aphasia. Aphasiology, 31(10), 1105–1126. 10.1080/02687038.2016.1239013

[bibr12-10870547251389329] Burd M. P. (2013). Talkativeness as a component of effective communication and impression management [Doctoral dissertation, University of California, San Diego]. eScholarship, University of California. https://escholarship.org/uc/item/08s265kd

[bibr13-10870547251389329] Calder S. D. Brennan-Jones C. G. Robinson M. Whitehouse A. Hill E. (2023). How we measure language skills of children at scale: A call to move beyond domain-specific tests as a proxy for language. International Journal of Speech-Language Pathology, 25(3), 440–448. 10.1080/17549507.2023.2171488 36786688

[bibr14-10870547251389329] Campbell M. , et al (2020). Synthesis without meta-analysis (SWiM) in systematic reviews: Reporting guideline. BMJ, 368, Article l6890. 10.1136/bmj.l6890 PMC7190266 31948937

[bibr15-10870547251389329] Carruthers S. Taylor L. Sadiq H. Tripp G. (2021). The profile of pragmatic language impairments in children with ADHD: A systematic review. Development and Psychopathology, 34(5), 1938–1960. 10.1017/S0954579421000328 33973504

[bibr16-10870547251389329] *Coelho R. M. Drummond C. Mota N. B. Erthal P. Bernardes G. Lima G. Molina R. Sudo F. K. Tannock R. Mattos P. (2021). Network analysis of narrative discourse and attention-deficit hyperactivity symptoms in adults. PLoS ONE, 16(4), e0245113. 10.1371/journal.pone.0245113 PMC8026017 33826632

[bibr17-10870547251389329] *Coelho R. M. Mattos P. Tannock R. (2018). Attention-deficit hyperactivity disorder (ADHD) and narrative discourse in older adults. Dementia & Neuropsychologia, 12(4), 374–379. 10.1590/1980-57642018dn12-040006 30546847 PMC6289475

[bibr18-10870547251389329] de Oliveira L. R. Brian J. Kelley E. Beal D. Nicolson R. Georgiades S. Iaboni A. Fragiadakis S. D. Ristic L. Anagnostou E. Sanjeevan T. (2020). Exploring the use of the verbal intelligence quotient as a proxy for language ability in autism spectrum disorder. Research in Autism Spectrum Disorders, 73, Article 101548. 10.1016/j.rasd.2020.101548

[bibr19-10870547251389329] *Derefinko K. J. Bailey U. L. Milich R. Lorch E. P. Riley E. (2009). The effects of stimulant medication on the online story narrations of children with ADHD. School Mental Health, 1, 171–182. 10.1007/s12310-009-9017-6

[bibr20-10870547251389329] Dipper L. T. Pritchard M . (2017). Discourse: Assessment and therapy. InTech. Advance online publication. 10.5772/intechopen.69894

[bibr21-10870547251389329] Ebert K. D. Scott C. M. (2014). Relationships between narrative language samples and norm-referenced test scores in language assessments of school-age children. Language, Speech, and Hearing Services in Schools, 45(4), 337–350. 10.1044/2014_LSHSS-14-0034 25104111

[bibr22-10870547251389329] *Engelhardt P.E. Ferreira F. Nigg J.T. (2011). Language production strategies and disfluencies in multi-clause network descriptions: A study of adult attention-deficit/hyperactivity disorder. Neuropsychology, 25(4), 442–453. 10.1037/a0022436 21463044 PMC5189676

[bibr23-10870547251389329] *Flake R. A. Lorch E. P. Milich R. (2007). The effects of thematic importance on story recall among children with attention deficit hyperactivity disorder and comparison children. Journal of Abnormal Child Psychology, 35(1), 43–53. 10.1007/s10802-006-9078-z 17136457

[bibr24-10870547251389329] *Flory K. Milich R. Lorch E. P. Hayden A. N. Strange C. Welsh R. (2006). Online story comprehension among children with ADHD: Which core deficits are involved? Journal of Abnormal Child Psychology, 34(6), 850–862. 10.1007/s10802-006-9070-7 17051434

[bibr25-10870547251389329] *Freer B. D. Hayden A. Lorch E. P. Milich R. (2011). The stories they tell: Story production difficulties of children with ADHD. School Psychology Review, 40(3), 352–366. 10.1080/02796015.2011.12087703

[bibr26-10870547251389329] Frizelle P. Thompson P. A. McDonald D. Bishop D. V. M. (2018). Growth in syntactic complexity between four years and adulthood: Evidence from a narrative task. Journal of Child Language, 45(5), 1174–1197. 10.1017/S0305000918000144 29860949

[bibr27-10870547251389329] Gillam R. Pearson N . (2004). The test of narrative language. Pro-Ed, Austin.

[bibr28-10870547251389329] Gillam R. B. Pearson N. A. (2017). Test of narrative language (TNL). Pro-Ed.

[bibr29-10870547251389329] Green B. C. Johnson K. A. Bretherton L. (2014). Pragmatic language difficulties in children with hyperactivity and attention problems: An integrated review. International Journal of Language & Communication Disorders, 49(1), 15–29. 10.1111/1460-6984.12056 24372883

[bibr30-10870547251389329] Hammill D. (1991). Detroit test of learning aptitude. Pro-Ed.

[bibr31-10870547251389329] *Hayden A. Lorch E. P. Milich R. Cosoreanu C. Van Neste J. (2018). Predictive inference generation and story comprehension among children with ADHD: Is making predictions helpful? Contemporary Educational Psychology, 53, 123–134. 10.1016/j.cedpsych.2018.02.003

[bibr32-10870547251389329] Higgins J. P. Thomas J. Chandler J. Cumpston M. Li T. Page M. J. Welch V. A. (Eds.). (2022). Cochrane handbook for systematic reviews of interventions (Version 6.3, updated February 2022). Cochrane. https://training.cochrane.org/handbook/archive/v6.3

[bibr33-10870547251389329] Hill E. Claessen M. Boyes M. Ward R. (2018). Discourse and cognition in speakers with acquired brain injury (ABI): A systematic review. International Journal of Language & Communication Disorders, 53(4), 689–717. 10.1111/1460-6984.12394 29781173

[bibr34-10870547251389329] Hill E. Claessen M. Whitworth A. Boyes M. (2021). Profiling variability and development of spoken discourse in mainstream adolescents. Clinical Linguistics & Phonetics, 35(2), 117–137. 10.1080/02699206.2020.1731607 32126850

[bibr35-10870547251389329] Hill E. Whitworth A. Boyes M. Ziegelaar M. Claessen M. (2021). The influence of genre on adolescent discourse skills: Do narratives tell the whole story? International Journal of Speech-Language Pathology, 23(5), 475–485. 10.1080/17549507.2020.1864016 33605172

[bibr36-10870547251389329] Hobbiss M. H. Lavie N. (2024). Sustained selective attention in adolescence: Cognitive development and predictors of distractibility at school. Journal of Experimental Child Psychology, 238, Article 105784. 10.1016/j.jecp.2023.105784 37862789

[bibr37-10870547251389329] Hobson H. Kalsi M. Cotton L. Forster M. Toseeb U. (2022). Supporting the mental health of children with speech, language and communication needs: The views and experiences of parents. Autism & Developmental Language Impairments, 7, 23969415221101137. 10.1177/23969415221101137 PMC9479119 36124076

[bibr38-10870547251389329] Horton W. S. Spieler D. H. (2007). Age-related differences in communication and audience design. Psychology and Aging, 22(2), 281–290. 10.1037/0882-7974.22.2.281 17563183

[bibr39-10870547251389329] *Houghton S. Cordin R. Durkin K. Whiting K. (2007). Salience and temporal sequencing of time-related actions in boys with attention deficit/hyperactivity disorder. Child Neuropsychology, 14(1), 60–70. 10.1080/09297040601160582 17852131

[bibr40-10870547251389329] *Jepsen I. B. Brynskov C. Thomsen P. H. Rask C. U. Jensen de López K. Lambek R. (2024). The role of language in the social and academic functioning of children with ADHD. Journal of Attention Disorders, 28(12), 1542–1554. 10.1177/10870547241266419 39077785

[bibr41-10870547251389329] Jepsen I. B. Hougaard E. Matthiesen S. T. Lambek R. (2022). A systematic review and meta-analysis of narrative language abilities in children with Attention-Deficit/Hyperactivity Disorder (ADHD). Research on Child and Adolescent Psychopathology, 50(6), 737–751. 10.1007/s10802-021-00871-4 34807333

[bibr42-10870547251389329] Johnson S. Lim E. Jacoby P. Faraone S. V. Su B. M. Solmi M. Forrest B. Furfaro B. von Klier K. Downs J. Chen W. (2025). Prevalence of attention deficit hyperactivity disorder/hyperkinetic disorder of pediatric and adult populations in clinical settings: A systematic review, meta-analysis and meta-regression. Molecular Psychiatry. Advance online publication. 10.1038/s41380-025-03178-8 PMC12700782 40877470

[bibr43-10870547251389329] Kemper S. (2006). Language in adulthood. In Bialystok E. Craik F. I. M. (Eds.), Lifespan cognition: Mechanisms of change (pp. 223–238). Oxford University Press. 10.1093/acprof:oso/9780195169539.003.0015

[bibr44-10870547251389329] Kmet L. M. Cook L. S. Lee R. C. (2004). Standard quality assessment criteria for evaluating primary research papers from a variety of fields. Alberta Heritage Foundation for Medical Research. 10.7939/R37M04F16

[bibr45-10870547251389329] Korkman M. Kirk U. Kemp S. (1998). NEPSY: A developmental neuropsychological assessment manual. The Psychological Corporation.

[bibr46-10870547251389329] Korrel H. Mueller K. L. Silk T. Anderson V. Sciberras E. (2017). Research review: Language problems in children with Attention-Deficit Hyperactivity Disorder - A systematic meta-analytic review. Journal of Child Psychology and Psychiatry, 58, 640–654. 10.1111/jcpp.12688 28186338

[bibr47-10870547251389329] *Kuijper S. J. Hartman C. A. Bogaerds-Hazenberg S. Hendriks P. (2017). Narrative production in children with Autism Spectrum Disorder (ASD) and children with Attention-Deficit/Hyperactivity Disorder (ADHD): Similarities and differences. Journal of Abnormal Psychology, 126(1), 63–75. 10.1037/abn0000231 27893232

[bibr48-10870547251389329] *Kuijper S. J. Hartman C. A. Hendriks P. (2015). Who is he? Children with ASD and ADHD take the listener into account in their production of ambiguous pronouns. PLOS ONE, 10(7), e0132408. 10.1371/journal.pone.0132408 PMC4492581 26147200

[bibr49-10870547251389329] Kysow K. Park J. Johnston C. (2017). The use of compensatory strategies in adults with ADHD symptoms. Attention Deficit and Hyperactivity Disorders, 9(2), 73–88. 10.1007/s12402-016-0205-6 27614892

[bibr50-10870547251389329] *Lee H. Sim H. Lee E. Choi D. (2017). Disfluency characteristics of children with attention-deficit/hyperactivity disorder symptoms. Journal of Communication Disorders, 65, 54–64. 10.1016/j.jcomdis.2016.12.001 28038762

[bibr51-10870547251389329] Li X. Hu D. Deng W. Tao Q. Hu Y. Yang X. Wang Z. Tao R. Yang L. Zhang X. (2017). Pragmatic ability deficit in schizophrenia and associated theory of mind and executive function. Frontiers in Psychology, 8, 2164–2175. 10.3389/fpsyg.2017.02164 29321753 PMC5732175

[bibr52-10870547251389329] Lindgren J. (2023). Age and task type effects on comprehension and production of narrative macrostructure: Storytelling and retelling by Swedish-speaking children aged 6 and 8. Frontiers in Communication, 8, Article 1252260. 10.3389/fcomm.2023.1252260

[bibr53-10870547251389329] *Lorch E. P. Diener M. B. Sanchez R. P. Milich R. Welsh R. van den Broek P. (1999). The effects of story structure on the recall of stories in children with attention deficit hyperactivity disorder. Journal of Educational Psychology, 91(2), 273–283. 10.1037/0022-0663.91.2.273

[bibr54-10870547251389329] *Lorch E. P. Milich R. Flake R. A. Ohlendorf J. Little S. (2010). A developmental examination of story recall and coherence among children with ADHD. Journal of Abnormal Child Psychology, 38, 291–301. 10.1007/s10802-009-9377-2 20024672 PMC4036495

[bibr55-10870547251389329] Lord C. Risi S. Lambrecht L. Cook E. H. Leventhal B. L. DiLavore P. C. Pickles A. Rutter M. (2000). The Autism Diagnostic Observation Schedule - Generic: A standard measure of social and communication deficits associated with the spectrum of autism. Journal of Autism and Developmental Disorders, 30, 205–223. 10.1023/A:1005592401947 11055457

[bibr56-10870547251389329] *Luo F. T. Timler G. R. (2008). Narrative organization skills in children with attention deficit hyperactivity disorder and language impairment: Application of the causal network model. Clinical Linguistics & Phonetics, 22(1), 25–46. 10.1080/02699200701627430 18092218

[bibr57-10870547251389329] Marini A. (2022). The beauty of diversity in cognitive neuroscience: The case of sex-related effects in language production networks. Journal of Neuroscience Research, 101(5), 633–642. 10.1002/jnr.25096 35692091

[bibr58-10870547251389329] Marini A. Petriglia F. D’Ortenzio S. Bosco F. M. Gasparotto G . (2025). Unveiling the dynamics of discourse production in healthy aging and its connection to cognitive skills. Discourse Processes, 62(6–7), 479–501. 10.1080/0163853X.2025.2507548

[bibr59-10870547251389329] *Mathers M. E. (2006). Aspects of language in children with ADHD: Applying functional analyses to explore language use. Journal of Attention Disorders, 9(3), 523–533. 10.1177/1087054705282437 16481669

[bibr60-10870547251389329] Méndez-Freije I. Areces D. Rodríguez C. (2023). Language skills in children with attention deficit hyperactivity disorder and developmental language disorder: A systematic review. Children, 11(1), 14. 10.3390/children11010014 38275435 PMC10814652

[bibr61-10870547251389329] *Miniscalco C. Hagberg B. Kadesjö B. Westerlund M. Gillberg C. (2007). Narrative skills, cognitive profiles and neuropsychiatric disorders in 7-8-year-old children with late developing language. International Journal of Language & Communication Disorders, 42(6), 665–681. 10.1080/13682820601084428 17852517

[bibr62-10870547251389329] Moher D. Liberati A. Tetzlaff J. Altman D. G. , & PRISMA Group. (2009). Preferred reporting items for systematic reviews and meta-analyses: The PRISMA statement. Annals of Internal Medicine, 151(4), 264–269. 10.7326/0003-4819-151-4-200908180-00135 19622511

[bibr63-10870547251389329] *Moonsamy S. Greenop K. Jordaan H. (2009). Cognitive processing and narrative discourse production in children with ADHD. South African Journal of Psychology, 39(3), 326–335. https://journals.co.za/doi/abs/10.10520/EJC98544

[bibr64-10870547251389329] Nilsen E. S. Mewhort Buist T. A. Gillis R. Fugelsang J. (2013). Communicative perspective-taking performance of adults with ADHD symptoms. Journal of Attention Disorders, 17(7), 589–597. 10.1177/1087054711428947 22298091

[bibr65-10870547251389329] *Nilsen E. S. Varghese A. Xu Z. Fecica A. (2015). Children with stronger executive functioning and fewer ADHD traits produce more effective referential statements. Cognitive Development, 36, 68–82. 10.1016/j.cogdev.2015.09.001

[bibr66-10870547251389329] Nippold M. A . (2007). Later language development : School-age children, adolescents, and young adults (3rd ed.). PRO-ED.

[bibr67-10870547251389329] Oram J. Fine J. Okamoto C. Tannock R. (1999). Assessing the language of children with attention deficit hyperactivity disorder. American Journal of Speech-Language Pathology, 8(1), 72–80. 10.1044/1058-0360.0801.72

[bibr68-10870547251389329] Page M. J. McKenzie J. E. Bossuyt P. M. Boutron I. Hoffmann T. C. Mulrow C. D. Shamseer L. Tetzlaff J. M. Akl E. A. Brennan S. E. Chou R. Glanville J. Grimshaw J. M. Hróbjartsson A. Lalu M. M. Li T. Loder E. W. Mayo-Wilson E. McDonald S. , . . . Moher D. (2021). The PRISMA 2020 statement: An updated guideline for reporting systematic reviews. BMJ, 372, n71. 10.1136/bmj.n71 PMC8005924 33782057

[bibr69-10870547251389329] *Papaeliou C. F. Maniadaki K. Kakouros E. (2015). Association between story recall and other language abilities in schoolchildren with ADHD. Journal of Attention Disorders, 19(1), 53–62. 10.1177/1087054712446812 22837548

[bibr70-10870547251389329] Pawełczyk A. Łojek E. Żurner N. Pawełczyk T. (2020). Pragmatic language dysfunctions in schizophrenia and depression patients: A preliminary study. Journal of Psychiatry and Clinical Psychology, 20(3), 3–12. 10.15557/PiPK.2020.0001

[bibr71-10870547251389329] Pineda-Alhucema W. Aristizabal E. Escudero-Cabarcas J. Acosta-López J. E. Vélez J. I. (2018). Executive function and theory of mind in children with ADHD: A systematic review. Neuropsychology Review, 28, 341–358. 10.1007/s11065-018-9381-9 30168020

[bibr72-10870547251389329] Polanczyk G. de Lima M. S. Horta B. L. Biederman J. Rohde L. A. (2007). The worldwide prevalence of ADHD: A systematic review and metaregression analysis. American Journal of Psychiatry, 164(6), 942–948. 10.1176/ajp.2007.164.6.942 17541055

[bibr73-10870547251389329] Polanin J. R. Pigott T. D. Espelage D. L. Grotpeter J. K. (2019). Best practice guidelines for abstract screening in large-evidence systematic reviews and meta-analyses. Research Synthesis Methods, 10(3), 330–342. 10.1002/jrsm.1354

[bibr74-10870547251389329] Power E. Weir S. Richardson J. Fromm D. Forbes M. Macwhinney B. Togher L. (2020). Patterns of narrative discourse in early recovery following severe traumatic brain injury. Brain Injury, 34(1), 98–109. 10.1080/02699052.2019.1682192 31661629 PMC8903041

[bibr75-10870547251389329] *Purvis K. L. Tannock R. (1997). Language abilities in children with attention deficit hyperactivity disorder, reading disabilities, and normal controls. Journal of Abnormal Child Psychology, 25, 133–144. 10.1023/A:1025731529006 9109030

[bibr76-10870547251389329] *Redmond S. M. (2004). Conversational profiles of children with ADHD, SLI and typical development. Clinical Linguistics & Phonetics, 18(2), 107–125. 10.1080/02699200310001611612 15086133

[bibr77-10870547251389329] *Redmond S. M. Ash A. C. Li H. Zhang Y. (2024). Links among attention-deficit/hyperactivity disorder symptoms and psycholinguistic abilities are different for children with and without developmental language disorder. American Journal of Speech-Language Pathology, 33(5), 2344–2363. 10.1044/2024_AJSLP-23-00388 38980144 PMC11427743

[bibr78-10870547251389329] *Redmond S. M. Ash A. C. Zhang Y. (2023). A preliminary study of the effects of stimulant medications on estimates of psycholinguistic abilities for children with attention-deficit/hyperactivity disorder. Clinical Linguistics & Phonetics, 38(10), 949–969. 10.1080/02699206.2023.2273750 37906703 PMC11058111

[bibr79-10870547251389329] *Redmond S. M. Thompson H. L. Goldstein S. (2011). Psycholinguistic profiling differentiates specific language impairment from typical development and from attention-deficit/hyperactivity disorder. Journal of Speech, Language, and Hearing Research, 54(1), 99–117. 10.1044/1092-4388(2010/10-0010) PMC4493886 20719871

[bibr80-10870547251389329] Renfrew C. (1995). The bus story: A test of narrative speech. ACER.

[bibr81-10870547251389329] Renfrew C . (1997). Bus story test : A test of narrative speech (4th ed.). Winslow.

[bibr82-10870547251389329] *Renz K. Lorch E. P. Milich R. Lemberger C. Bodner A. Welsh R. (2003). On-line story representation in boys with attention deficit hyperactivity disorder. Journal of Abnormal Child Psychology, 31, 93–104. 10.1023/A:1021777417160 12597702

[bibr83-10870547251389329] *Rumpf A. L. Kamp-Becker I. Becker K. Kauschke C. (2012). Narrative competence and internal state language of children with Asperger syndrome and ADHD. Research in Developmental Disabilities, 33(5), 1395–1407. 10.1016/j.ridd.2012.03.007 22522198

[bibr84-10870547251389329] Sato M. (2020). The neurobiology of sex differences during language processing in healthy adults: A systematic review and a meta-analysis. Neuropsychologia, 140, Article 107404. 10.1016/j.neuropsychologia.2020.107404 32087207

[bibr85-10870547251389329] Shroff D. M. Dunn N. C. Green C. D. Breaux R. Becker S. P. Langberg J. M. (2024). Predictors of executive function trajectories in adolescents with and without ADHD: Links with academic outcomes. Development and Psychopathology, 36(3), 1489–1502. 10.1017/S0954579423000743 37434496

[bibr86-10870547251389329] Song P. Zha M. Yang Q. Zhang Y. Li X. Rudan I. (2021). The prevalence of adult attention-deficit hyperactivity disorder: A global systematic review and meta-analysis. Journal of Global Health, 11, e04009. 10.7189/jogh.11.04009 PMC7916320 33692893

[bibr87-10870547251389329] Spencer T. D. Petersen D. B. (2020). Narrative intervention: Principles to practice. Language, Speech, and Hearing Services in Schools, 51(4), 1081–1096. 10.1044/2020_LSHSS-20-00015 32776816

[bibr88-10870547251389329] *Staikova E. Gomes H. Tartter V. McCabe A. Halperin J. M. (2013). Pragmatic deficits and social impairment in children with ADHD. Journal of Child Psychology and Psychiatry, 54, 1275–1283. 10.1111/jcpp.12082 23682627 PMC3648855

[bibr89-10870547251389329] Stein N. L. Glenn C. G . (1979). An analysis of story comprehension in elementary school children. In Freedle R. O. (Ed.), New directions in discourse processing (pp. 53–120). Ablex.

[bibr90-10870547251389329] Tannock R. Schachar R. (1996). Executive dysfunction as an underlying mechanism of behavior and language problems in attention deficit hyperactivity disorder. In Beitchman J. H. Cohen N. J. Konstantareas M. M. Tannock R. (Eds.), Language, learning, and behavior disorders: Developmental, biological, and clinical perspectives (pp. 128–155). Cambridge University Press.

[bibr91-10870547251389329] Tatar Z. B. Cansız A. (2022). Executive function deficits contribute to poor theory of mind abilities in adults with ADHD. Applied Neuropsychology: Adult, 29(2), 244–251. 10.1080/23279095.2020.1736074 32186409

[bibr92-10870547251389329] Taylor C. del Fante D. (2020). Comparing across languages in corpus and discourse analysis: Some issues and approaches. Meta, 65(1), 29–50. 10.7202/1073635ar

[bibr93-10870547251389329] *Timler G. R. White K. E. (2014). Social communication assessment and intervention for children with attention problems. In Hwa-Froelich D. A. (Ed.), Social communication development and disorders (pp. 252–286). Psychology Press.

[bibr94-10870547251389329] Turkstra L. S . (2000). Should my shirt be tucked in or left out? The communication context of adolescence. Aphasiology, 14(4), 349–364. 10.1080/026870300401405

[bibr95-10870547251389329] *van Lambalgen M. van Kruistum C. Parigger E. (2008). Discourse processing in Attention-Deficit Hyperactivity Disorder (ADHD). Journal of Logic, Language and Information, 17, 467–487. 10.1007/s10849-008-9066-5

[bibr96-10870547251389329] *Van Neste J. Hayden A. Lorch E. P. Milich R. (2015). Inference generation and story comprehension among children with ADHD. Journal of Abnormal Child Psychology, 43, 259–270. 10.1007/s10802-014-9899-0 24969853

[bibr97-10870547251389329] Weiss L. G. Saklofske D. H. (2020). Mediators of IQ test score differences across racial and ethnic groups: The case for environmental and social justice. Personality and Individual Differences, 161, Article 109962. 10.1016/j.paid.2020.109962

[bibr98-10870547251389329] Westerveld M. F. Claessen M. (2014). Clinician survey of language sampling practices in Australia. International Journal of Speech-Language Pathology, 16(3), 242–249. 10.3109/17549507.2013.871336 24447161

[bibr99-10870547251389329] Whitworth A. Claessen M. Leitão S. Webster J. (2015). Beyond narrative: Is there an implicit structure to the way in which adults organise their discourse? Clinical Linguistics & Phonetics, 29(6), 455–481. 10.3109/02699206.2015.1020450 25774761

[bibr100-10870547251389329] Wiig E. H. Semel E. Secord W. A. (2013). Clinical evaluation of language fundamentals-Fifth edition (CELF-5). NCS Pearson.

[bibr101-10870547251389329] Zenaro M. P. Rossi N. F. Souza A. L. D. M. D. Giacheti C. M. (2019). Oral narrative structure and coherence of children with attention deficit hyperactivity disorder. Communication Disorders, Audiology and Swallowing, 31(6), e1–e8. 10.1590/2317-1782/20192018197 31778423

[bibr102-10870547251389329] Zentall S. S. (1988). Production deficiencies in elicited language but not in the spontaneous verbalizations of hyperactive children. Journal of Abnormal Child Psychology, 16, 657–673. 10.1007/BF00913476 3216074

