# Supplementary material for: Narrative and Non-Narrative Discourse Skills in ADHD Across the Lifespan: A Systematic Review of the Literature
Source: J Atten Disord. 2025 Nov 18;30(5):629–48. doi: 10.1177/10870547251389329 (PMC13033048; doi:10.1177/10870547251389329)
Supplement: sj-docx-1-jad-10.1177_10870547251389329 – Supplemental material for Narrative and Non-Narrative Discourse Skills in ADHD Across the Lifespan: A Systematic Review of the Literature [file sj-docx-1-jad-10.1177_10870547251389329.docx]

**Supplementary Material**

**Description of four-level analysis framework**

**Micro-linguistic measures** capture productivity (total number of words or sentences), grammatical and sentence complexity, the diversity of vocabulary, and language fluency (e.g., excessive use of “um”, “uh”, filler words, filled pauses, repetitions would signify dysfluency).

**Macro-linguistic** **measures** capture the degree to which speakers use appropriate cohesive devices to connect people, ideas, and/or events, between sentences. These devices include referential ties (e.g., pronouns, ‘he’, ‘him’), demonstratives (e.g., ‘here’, ‘there’), and conjunctions (e.g., ‘and’, ‘because’) (Halliday and Hasan, 2014).

**Macro-structural measures** capture the coherence (local and global), relevance, and informativeness of discourse output (Power et al., 2020). Local coherence refers to the logical flow of ideas from once sentence to the next, whereas global coherence denotes refers to the relevance of the idea presented by each sentence to the overall topic of discourse (i.e., global coherence is sensitive to atypical digression) (Glosser and Deser, 1991).

**Super-structural measures** capture the overall structure of a speaker’s discourse output. For example, in storytelling, superstructural analysis could evaluate whether a speaker has included all the stereotypical elements such as the beginning, climax, and resolution in a logical manner (Hill et al., 2021; Power et al., 2020; Stein and Glenn, 1979). In non-narrative genres, a definition and series of categorised facts (or ‘evidence’), and a conclusion are expected in expository discourse (e.g., a definition of ‘obesity’ and information related to the ‘causes’ and ‘consequences’ of the disease).

**References**

Glosser G and Deser T (1991) Patterns of discourse production among neurological patients with fluent language disorders. *Brain and Language* 40: 67-88.

Halliday M and Hasan R (2014) *Cohesion in English*. London: Routledge.

Hill E, Claessen M, Whitworth A, et al. (2021) Profiling variability and development of spoken discourse in mainstream adolescents. *Clinical Linguistics and Phonetics* 35: 117-137.

Power E, Weir S, Richardson J, et al. (2020) Patterns of narrative discourse in early recovery following severe traumatic brain injury. *Brain Injury*, 34: 98-109.

Stein N and Glenn C (1979) An analysis of story comprehension in elementary school children. In: Freedle R (ed) *Advances in Discourse Processing (Vol. 2): New directions in discourse processing.* Norwood, NJ: Ablex, pp.53-120
