# Supplementary material for: Narrative and Non-Narrative Discourse Skills in ADHD Across the Lifespan: A Systematic Review of the Literature
Source: J Atten Disord. 2025 Nov 18;30(5):629–48. doi: 10.1177/10870547251389329 (PMC13033048; doi:10.1177/10870547251389329)
Supplement: sj-docx-2-jad-10.1177_10870547251389329 – Supplemental material for Narrative and Non-Narrative Discourse Skills in ADHD Across the Lifespan: A Systematic Review of the Literature [file sj-docx-2-jad-10.1177_10870547251389329.docx]

| **Supplementary Table 1**  Methodological quality assessment across included studies (Kmet et al., 2004) | | | | | | | | | | | | | | | | | | | | | | | | | | | | | | | | | | | | | | | | |
| --- | --- | --- | --- | --- | --- | --- | --- | --- | --- | --- | --- | --- | --- | --- | --- | --- | --- | --- | --- | --- | --- | --- | --- | --- | --- | --- | --- | --- | --- | --- | --- | --- | --- | --- | --- | --- | --- | --- | --- | --- |
| **Item** | Baixauli Fortea et al., 2018 | Bangert & Finestack, 2020 | Barkley et al., 1983 | Bergman & Hallin, 2021 | Boo, 2022 | Coelho et al., 2018 | Coelho et al., 2021 | Derefinko et al., 2009 | Engelhardt et al., 2011 | Flake et al., 2007 | Flory et al., 2006 | Freer et al., 2011 | Hayden et al., 2018 | Houghton et al., 2008 | Jepsen et al., 2024 | Kuijper et al., 2015 | Kuijper et al., 2017 | Lee et al., 2017 | Lorch et al., 1999 | Lorch et al., 2010 | Luo & Timler, 2008 | Mathers, 2006 | Maniscalco et al., 2007 | Moonsamy et al., 2009 | Nilsen et al., 2015 | Papaeliou et al., 2015 | Purvis & Tannock, 1997 | Redmond, 2004 | Redmond, 2011 | Redmond et al., 2023 | Redmond et al., 2024 | Renz et al., 2003 | Rumf et al., 2012 | Staikova et al., 2013 | Timler & White, 2014 | van Lambalgen et al., 2008 | Van Neste et al., 2015 | Zenaro et al., 2019 | Zentall, 1988 |  |
| 1. Question/Aim | 2 | 2 | 2 | 2 | 1 | 1 | 2 | 2 | 2 | 2 | 2 | 2 | 2 | 2 | 2 | 2 | 2 | 2 | 2 | 2 | 2 | 1 | 2 | 2 | 2 | 1 | 2 | 2 | 2 | 2 | 2 | 1 | 2 | 1 | 2 | 2 | 2 | 2 | 2 |  |
| 1. Study design | 2 | 2 | 2 | 2 | 2 | 2 | 2 | 2 | 2 | 1 | 2 | 2 | 2 | 2 | 2 | 2 | 2 | 2 | 2 | 2 | 2 | 2 | 2 | 2 | 2 | 2 | 1 | 1 | 1 | 2 | 2 | 1 | 1 | 2 | 2 | 2 | 2 | 2 | 2 |  |
| 1. Subject/Input selection | 2 | 1 | 2 | 2 | 1 | 1 | 2 | 1 | 2 | 1 | 1 | 1 | 2 | 1 | 2 | 1 | 1 | 1 | 1 | 2 | 1 | 1 | 2 | 2 | 1 | 2 | 1 | 2 | 2 | 1 | 2 | 2 | 1 | 1 | 2 | 1 | 1 | 2 | 1 |  |
| 1. Subject characteristics | 2 | 2 | 1 | 2 | 2 | 1 | 2 | 2 | 2 | 2 | 2 | 1 | 2 | 2 | 2 | 2 | 2 | 1 | 2 | 2 | 2 | 1 | 1 | 1 | 1 | 2 | 1 | 2 | 2 | 2 | 2 | 2 | 2 | 2 | 2 | 1 | 2 | 1 | 2 |  |
| 1. Randomisation description | - | - | 2 | - | - | - | - | 2 | - | - | - | - | - | - | - | - | - | - | - | - | - | - | - | - | - | - | - | - | - | - | - | - | - | - | - | - | - | - | - |  |
| 1. Investigator blinding | - | - | 2 | - | - | - | - | 2 | - | - | - | - | - | - | - | - | - | - | - | - | - | - | - | - | - | - | - | - | - | - | - | - | - | - | - | - | - | - | - |  |
| 1. Subject blinding | - | - | 2 | - | - | - | - | 1 | - | - | - | - | - | - | - | - | - | - | - | - | - | - | - | - | - | - | - | - | - | - | - | - | - | - | - | - | - | - | - |  |
| 1. Outcome definition | 2 | 2 | 2 | 2 | 2 | 1 | 2 | 2 | 2 | 2 | 2 | 2 | 2 | 2 | 2 | 1 | 2 | 2 | 2 | 2 | 2 | 2 | 2 | 2 | 2 | 2 | 2 | 2 | 2 | 2 | 2 | 2 | 1 | 2 | 2 | 2 | 1 | 1 | 1 |  |
| 1. Sample size | 2 | 1 | 1 | 2 | 2 | 2 | 2 | 2 | 2 | 2 | 2 | 2 | 2 | 2 | 2 | 1 | 2 | 2 | 2 | 2 | 2 | 2 | 2 | 1 | 2 | 2 | 1 | 2 | 2 | 2 | 2 | 2 | 2 | 1 | 1 | 1 | 2 | 2 | 1 |  |
| 1. Analytic methods | 2 | 2 | 2 | 2 | 2 | 2 | 2 | 2 | 2 | 2 | 2 | 2 | 2 | 2 | 2 | 2 | 2 | 2 | 2 | 2 | 2 | 2 | 1 | 2 | 2 | 2 | 2 | 2 | 2 | 2 | 2 | 2 | 2 | 3 | 2 | 1 | 2 | 2 | 2 |  |
| 1. Variance estimates | 2 | 2 | 2 | 2 | 2 | - | 0 | 2 | 2 | 2 | 2 | 2 | 2 | 2 | 2 | 2 | 2 | 2 | 2 | 2 | 2 | 2 | 2 | 2 | 2 | 2 | 2 | 2 | 2 | 2 | 2 | 2 | 2 | 2 | 2 | 1 | 2 | 1 | 2 |  |
| 1. Confounding control | 1 | 1 | 1 | 2 | 2 | 1 | 2 | 1 | 1 | 1 | 2 | 2 | 2 | 2 | 2 | 2 | 2 | 2 | 2 | 2 | 2 | 2 | 1 | 1 | 2 | 2 | 2 | 2 | 1 | 2 | 2 | 1 | 1 | 2 | 2 | 1 | 2 | 2 | 1 |  |
| 1. Results detail | 2 | 2 | 2 | 2 | 2 | 2 | 1 | 2 | 2 | 2 | 2 | 2 | 2 | 2 | 2 | 2 | 2 | 2 | 2 | 2 | 2 | 1 | 2 | 2 | 2 | 2 | 2 | 2 | 2 | 1 | 2 | 2 | 2 | 2 | 2 | 2 | 2 | 2 | 2 |  |
| 1. Supported conclusions | 2 | 1 | 2 | 2 | 2 | 2 | 0 | 2 | 2 | 1 | 2 | 2 | 2 | 2 | 2 | 1 | 2 | 2 | 2 | 2 | 2 | 2 | 2 | 2 | 2 | 2 | 2 | 2 | 2 | 2 | 2 | 2 | 2 | 2 | 2 | 2 | 2 | 1 | 2 |  |
| **Total raw** | **21** | **18** | **25*** | **22** | **20** | **15** | **17** | **25*** | **21** | **18** | **21** | **20** | **22** | **21** | **22** | **18** | **21** | **20** | **21** | **22** | **21** | **18** | **19** | **19** | **20** | **21** | **18** | **21** | **20** | **20** | **22** | **19** | **18** | **20** | **21** | **16** | **20** | **18** | **18** |  |
| **Percentage^†^** | 95 | 82 | 89 | 100 | 91 | 75 | 77 | 89 | 95 | 82 | 95 | 91 | 100 | 95 | 100 | 82 | 95 | 91 | 95 | 100 | 95 | 82 | 86 | 86 | 91 | 95 | 82 | 95 | 91 | 91 | 100 | 86 | 82 | 91 | 95 | 73 | 91 | 82 | 82 |  |

*Note.* 2 = ‘Yes’, 1 = “Partial’, 0 = ‘No’, - = N/A (see Kmet et al., 2004). **^†^** indicates strength of quality where <50% = limited, 50-69% = adequate, 70-89% = strong, >90% = very strong (Kmet et al., 2004). * Indicates an intervention study, where scoring has been adjusted accordingly (Kmet et al., 2004).
